# A Small Compound Targeting Prohibitin with Potential Interest for Cognitive Deficit Rescue in Aging mice and Tau Pathology Treatment

**DOI:** 10.1038/s41598-020-57560-3

**Published:** 2020-01-24

**Authors:** Anne-Cécile Guyot, Charlotte Leuxe, Clémence Disdier, Nassima Oumata, Narciso Costa, Gwenaëlle Le Roux, Paloma Fernandez-Varela, Arnaud Duchon, Jean Baptiste Charbonnier, Yann Herault, Serena Pavoni, Hervé Galons, Emile Andriambeloson, Stéphanie Wagner, Laurent Meijer, Amie K. Lund, Aloïse Mabondzo

**Affiliations:** 1 Service de Pharmacologie et d’Immunoanalyse, CEA, Université Paris-Saclay, F-91191 Gif-sur Yvette, France; 2 Department of Pediatrics, Women & Infants Hospital of Rhode Island, The Warren Alpert Medical School, Brown University, Providence, RI USA; 3 University Paris Descartes, INSERM U1022, 4, avenue de l’Observatoire, 75006 Paris, France; 4 Institute for Integrative Biology of the Cell: Department of Biochemistry, Biophysics and Structural Biology,CEA, Université Paris-Saclay, F-91191 Gif-sur Yvette, France; 5 Université de Strasbourg, CNRS, INSERM, IGBMC, 1 rue Laurent Fries, 67404 Illkirch, France; 6 Service d’Etude des Prions et Infections Atypiques (SEPIA), Institut François Jacob, CEA, Université Paris-Saclay, Fontenay-aux-Roses, France; 7 Neurofit, 67400 Illkirch, France; 8 Perha-pharma, Centre de Perharidy, Roscoff, France; 9 Department of Biological Sciences, Advanced Environmental Research Institute, University of North Texas, Denton, TX USA

**Keywords:** Cognitive neuroscience, Neurogenesis, Target identification, Pharmacology, Pharmacodynamics, Pharmacokinetics, Drug discovery, Neuroscience

## Abstract

Neurodegenerative diseases, including Alzheimer’s and Parkinson’s disease, are characterized by increased protein aggregation in the brain, progressive neuronal loss, increased inflammation, and neurogenesis impairment. We analyzed the effects of a new purine derivative drug, PDD005, in attenuating mechanisms involved in the pathogenesis of neurodegenerative diseases, using both *in vivo* and *in vitro* models. We show that PDD005 is distributed to the brain and can rescue cognitive deficits associated with aging in mice. Treatment with PDD005 prevents impairment of neurogenesis by increasing sex-determining region Y-box 2, nestin, and also enhances synaptic function through upregulation of synaptophysin and postsynaptic density protein 95. PDD005 treatment also reduced neuro-inflammation by decreasing interleukin-1β expression, activation of astrocytes, and microglia. We identified prohibitin as a potential target in mediating the therapeutic effects of PDD005 for the treatment of cognitive deficit in aging mice. Additionally, in the current study, glycogen synthase kinase appears to attenuate tau pathology.

## Introduction

Based on current statistics, disorders of the central nervous system (CNS) will become a significant healthcare issue in the 21st century. Neurodegenerative disorders (NDs) can have a substantial impact on the quality of life for patients, often associated with progressive debilitation. Alzheimer’s disease and Parkinson’s disease are the most common forms of NDs for which the principal risk factor is age^1^. Current therapeutic strategies often treat only the symptoms of the disease and can have problematic side effects in some patients. NDs are characterized by aggregation and accumulation of cellular proteins^2^. For instance, in Alzheimer’s disease, cellular metabolism dysfunction in the CNS results in the accumulation of amyloid β (Aβ) peptides (Aβ-42 and Aβ-40) and hyperphosphorylation of tau protein, which are associated with selective loss of neurons in the brain^3^. Abnormal protein aggregation in the brain is also associated with inflammation^4^ and impaired neurogenesis^5^. A recent study reported that adult neurogenesis occurs in the dentate gyrus of the hippocampus throughout adulthood and is associated with plasticity, learning, and memory^6^. Neurogenesis has been shown to decline with normal aging^6^ and is reported to be decreased in Alzheimer’s and Parkinson’s diseases^7–10^. As such, both inflammation and impairment of adult neurogenesis have become a hallmark of NDs. Thus, harnessing inflammatory responses through targeted modulation of innate immunity may have potential therapeutic implications in driving endogenous neurogenesis and repair mechanisms in NDs^8^.

Purines are the building blocks of nucleic acids. They also serve as energy cofactors, components of coenzymes, and second messengers in many intracellular signal transduction processes. Originally, purine derivative drugs (PDDs) were discovered through an effort to screen for compounds with kinase inhibitor activity. Recent reports suggest that PDDs may have therapeutic potential in the treatment of pathological conditions such as NDs. For example, Oumata *et al*. reported that Roscovitine-derived compounds inhibit cyclin kinases and casein kinases 1, which are involved in the production of Aβ proteins and hyperphosphorylation of tau protein in Alzheimer’s disease^11^. Additional studies have demonstrated that purine derivatives promote the formation of amyloid precursor protein gene, and also stimulate the synthesis and secretion of synaptophysin (SYP), which is reported to be decreased in patients with Alzheimer’s disease. Purine derivatives modulate the Aβ proteins through inhibition of glycogen synthase (GSK)-3 activity and also alterations in γ-secretase activity (WO 2004/016612 and WO 2008/122767)^12^. However, to date, only the effects of PDD therapies on the amyloid signaling mechanism have been characterized, and as such, their potential for targeting multiple signaling pathways involved in NDs has not yet been determined.

An activated immune response in the brain has emerged as an important contributor to the etiology of NDs. As such, pharmacotherapies will likely require a multi-targeted approach to treat NDs and other neuroinflammatory disorders successfully. In the present study, we investigated the therapeutic outcomes of treatment with a PDD, which has a tertiary amine on the N6 position, PDD005, for cognitive deficit rescue in aging mice and tau pathology treatment. Additionally, we investigated the beneficial effects of PDD005 on synaptic function, neuroplasticity decline, neuroinflammation, and tau phosphorylation in aging mice. To elucidate a potential target, we also investigated the impact of PDD005-treatment on the expression of prohibitin (PHB), a neuroprotective protein, and modulator of mitochondrial function^13,14^ in aging mice using organotypic hippocampal slice cultures of a triple-transgenic mouse model (3xTg-AD).

## Results

### Assessment of translocation of PDD005 into the brain

*In vitro* and *in vivo* experiments were carried out to assess the translocation of PDD005 across the blood-brain barrier (BBB). First, we utilized a well-characterized cell-based BBB co-culture model^15^ to investigate the translocation properties of PDD005 *in vitro*. BBB integrity, due to proper tight junction (TJ) protein formation, is confirmed once ^14^C-sucrose measurement permeability (P_app_) is measured under 3.28 ± 0.82 × 10^−6^ cm.s^−1^. After demonstrating the integrity of the endothelial cell monolayer in the co-culture, we investigated the ability of PDD005 to cross the (apical) endothelial layer into the basal compartment, which is indicative of transport across the BBB into the brain. PDD005 was quantified in the apical and basolateral compartment after 60 min of exposure using liquid chromatography-mass spectrometry in tandem (LC-MS/MS). We demonstrate that PDD005 crosses the BBB with the apparent permeability value of about 35 × 10^−6^ cm.s^−1^. The P_app_ of PDD005 is similar to the P_app_ value of Memantine, which is another pharmaceutical used in the treatment of Alzheimer’s disease.

To analyze PDD005 translocation into the CNS *in vivo*, PDD005 was administered to wild-type (WT) young adult mice using either: (1) a single intraperitoneal injection (i.p.) (10 mg/kg), (2) a single oral administration (PO) (5 mg/kg), or (3) a subchronic subcutaneous (SC) administration (28 days, 30 mg/kg/days). The drug concentration was quantified in the both the brain and plasma by LC-MS/MS. The pharmacokinetic (PK) studies show that PDD005 can translocate into the brain tissue, even after a single PO or i.p. dose (Table S3). The maximum concentration (Cmax) of PDD005 in the plasma was 3.89 µM and 22.56 µM for oral and i.p. administration, respectively (Fig. S2A,C). The elimination half-life (t_1/2_) was about 1.9 h and 1.2 h for oral and i.p. administration, respectively. The C_max_ in the brain after administration was 44.93 nM for PO and 807.41 nM for i.p. administration (Fig. S2B,D). The brain/plasma partition coefficient (K_p brain_/_plasma_) at 6 h after administration was about 0.014 for oral and 0.033 for i.p. administration.

Subchronic SC administration of PDD005 (28 days, 30 mg/kg/day) increased PDD005 plasma concentrations (Fig. S2E) in mice. At the end of the 28 day treatment period, the K_p_ brain/plasma was about 0.44, suggesting brain entry of PDD005 after SC subchronic administration (Fig. S2F).

### PDD005 enhances behavioral performances in young adult mice and improves cognitive decline observed with aging

**Figure 1:**
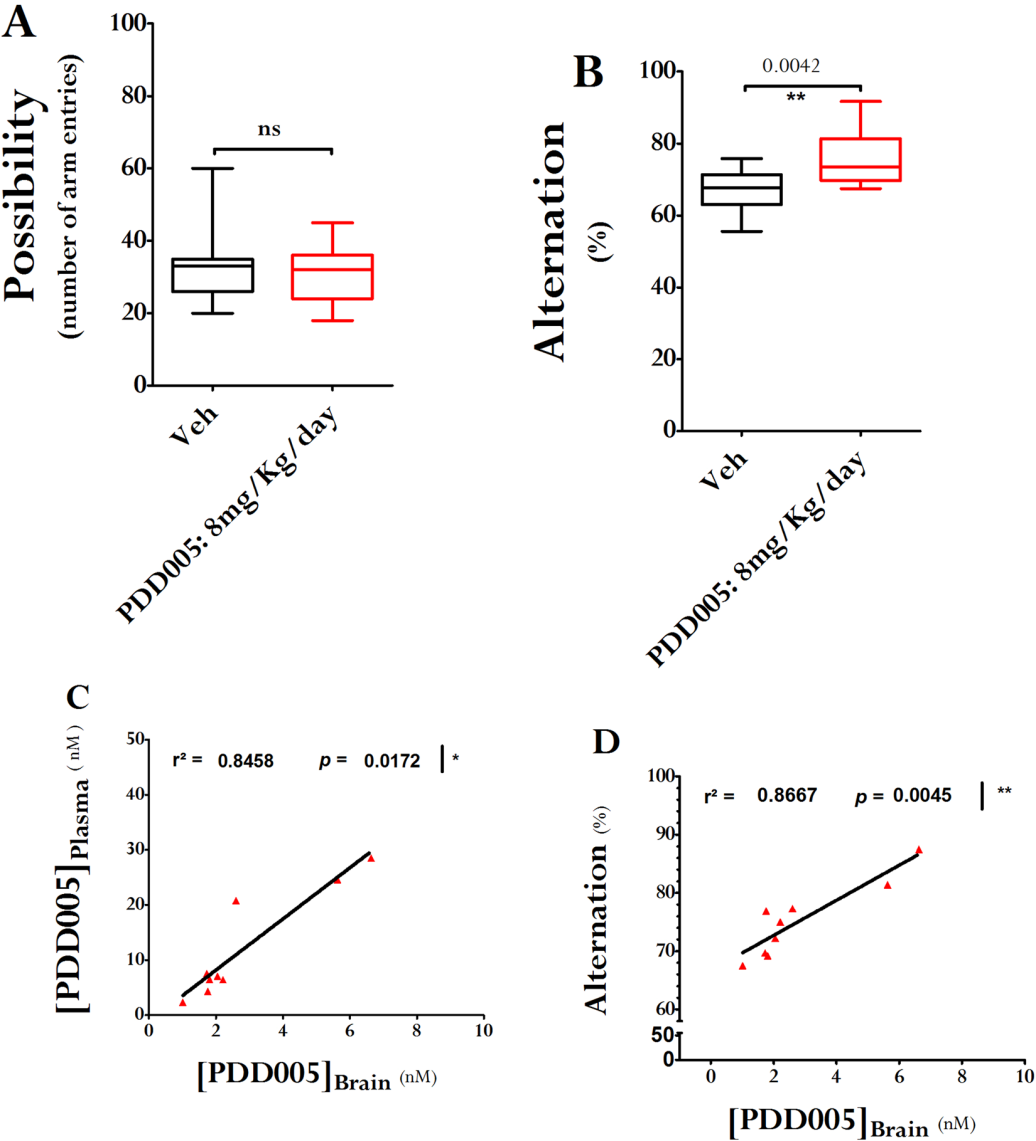
PDD005 improves cognitive performance in young adult mice. Young adult WT mice were exposed by SC injection to PDD005 at 8 mg/kg/day or vehicle for 28 days. (**A**,**B)** Plots illustrating the effect of PDD005 on short-term memory (working memory) in the Y-maze 3–4 weeks after the end of treatment. (**A**) General activity was estimated by counting the number of arm entries. PDD005 exposition has not shown to induce hyper or hypoactivity. Cognitive abilities were assessed by the percentage of alternation (**B**). Mann-Whitney tests were performed and **P < 0.01 indicates significant differences between PDD005 and control conditions. Data expressed as means ± SEM with n = 15 mice/condition. **(C)** Linear regression analysis shows a positive correlation between brain and plasma concentration of PDD005 at 4 weeks at the end of the exposure period. **(D)** Linear regression analysis shows a positive correlation between brain concentration of PDD005 and short-term memory abilities at 4 weeks after the end of the treatment. Spearman tests were performed and *P < 0.05 and **P < 0.001 indicate significant correlation. Data expressed as means ± SEM with n = 9 mice/condition.

First, we examined the impact of subchronic administration of PDD005 on long-term spatial memory in young adult WT mice. PDD005 (8 mg/kg/day) was administered via mini osmotic pumps continuously for a period of 28 days, and then the Y-maze test was performed. PDD005 -treated mice were observed to make less errors in the Y maze, and had a better spontaneous alternation score, compared to control mice (Fig. 1A,B). Additionally, we demonstrated that at this dose PDD005 is distributed to the brain, and the brain and plasma concentrations positively correlate (Fig. 1C; P = 0.0172). We also show a strong correlation between the concentration of PDD005 in the brain and working memory performance, as evaluated by the Y-maze test (Fig. 1D; P = 0.0045).

**Figure 2:**
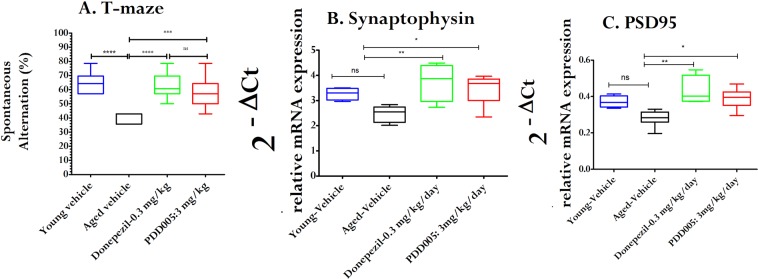
PDD005 improves cognitive performance in aging mice. Aging WT mice were exposed by i.p. administration to PDD005 at 3 mg/kg/day or vehicle for 28 days. (**A**) Graph showing that PDD005 rescues cognitive deficit in aging mice 3–4 weeks after treatment. Cognitive abilities were assessed by means of the percentage of alternation in the T-maze test. One-way ANOVA with the Tukey’s multiple comparison test for post hoc analysis were performed and ***P < 0.001 indicates significant differences between PDD005 and aged vehicle group. Data expressed as means ± SEM with n = 10 mice/condition. **(B,C)** Plots showing the effect of PDD005 on relative mRNA expression levels of *SYP* (**B**) and *PSD95* (**C**) in aging mice treated subchronically with PDD005. Transcriptional expression was quantified by RT-PCR and expressed as the relative expression of specific genes normalized to the housekeeping gene *HPRT* (2^−ΔCt^). One-way ANOVA and Tukey’s multiple comparison test for post hoc analysis were performed. *P < 0.05 and **P < 0.01 indicate significant differences between PDD005 and the aged vehicle group. Data expressed as means ± SEM with n = 4–6 mice/condition.

Cognitive impairment and dementia are disabling conditions that are increasingly common in an aging population. The T-maze continuous alternation task is a method implemented to evaluate spatial exploratory performance in mice. When used in aged mice, this paradigm allows for the demonstration of cognitive/memory deficits, and thus provides a useful model for screening compounds with cognitive-enhancing properties. We assessed cognitive deficits in aging WT mice with PDD005-treatment (i.p. 3 mg/kg/day for 28 days), compared to vehicle treatment (controls) using the T-maze test (Fig. 2A). The results demonstrate a cognitive decline with age (spontaneous alternation in the arm was 64.28 ± 5.38% in the young adult vs 40 ± 3.43% in the aging group, P < 0.0001). However, PDD005-treatment resulted in an apparent improvement in cognitive decline, bringing the abilities of aged mice treated with PDD005 to similar levels as that observed in the young adult mice. Donepezil, an FDA approved drug used in the treatment of dementia and Alzheimer’s disease, was used as a positive control and to evaluate the efficacy of PDD005. In terms of mitigating cognitive decline, both Donepezil and PDD005 appeared to have similar outcomes in our study. These same experiments were also conducted in two other disease models, (1) lipopolysaccharide (LPS)-treated mice, which induces neuro-inflammation; and (2) scopolamine-treated mice, which is known to produce memory deficits in rodent models. In agreement with our initial findings, PDD005-treatement improved cognitive deficit in both models (Figs. S3 and S4).

### PDD005 enhances synaptic markers in the aging brain

We next investigated whether the improvement of long-term spatial memory in aging WT mice was associated with increased synaptic function by examining transcriptional regulation of synaptophysin (*SYP*) and postsynaptic density protein 95 (*PSD95*). The cognitive decline observed in aging mice is associated with decreased synaptic markers, as shown by decreased transcriptional expression of *SYP* and *PSD95* with age. Brains from aging mice treated with PDD005 (i.p., 3 or 30 mg/kg/day for 28 days) exhibited increased *SYP* (P < 0.05) and *PSD95* (P < 0.05) mRNA transcript expression. The levels of expression of the two markers in PDD005-treated aged mice were comparable to the expression observed in the young adult mice (Fig. 2B,C). Thus, the improvement of cognitive function in the PDD005-treated aged is associated with increased expression of *SYP* and *PSD95*.

### Neuroplasticity decline can be rescued with PDD005 treatment

**Figure 3:**
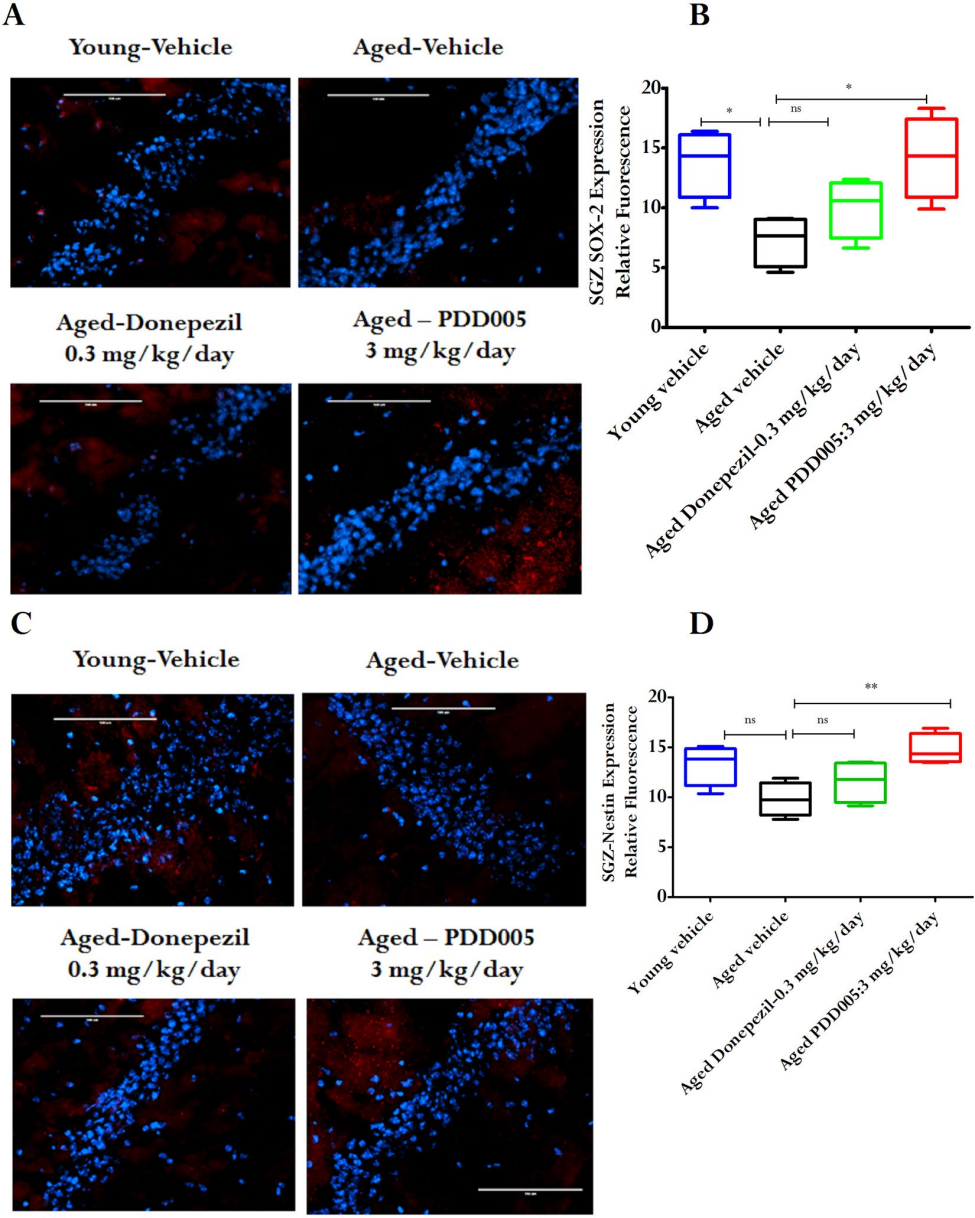
PDD005 reduces decline in neuroplasticity in aging mice. Aged mice showed a reduction in SOX-2 and nestin expression compared to young mice in the subgranular zone (SGZ). This can be alleviated by PDD005. (**A)** Images showing an example of SOX-2 (red) expression in the SGZ of the dentate gyrus of the hippocampus. Scale bar = 100 μm. (**B)** Graphs representing the average relative fluorescence of SOX-2 (red) in the SGZ for each group. (**C)** Images showing an example of nestin (red) expression in the SGZ of the hippocampus. (**D)** Graph representing the average relative fluorescence of nestin (red) in the SGZ for each group. Scale bar = 100 μm. Significant differences determined by using one-way ANOVA with Tukey’s test. *P < 0.05 compared to aged-vehicle. All error bars indicate SEM, n = 4 mice/condition.

SOX-2 (Sex determining Region Y-box 2) is a transcriptional factor that appears to play a protective role by mediating neurogenesis^16^. Moreover, SOX-2-labeled quiescent stem cells have been reported to decline with aging^6^. Nestin and SOX-2 co-expression is associated with actively increasing non-radial type 2 neural stem cells, which is used as proliferative marker to evaluate neuroplasticity^6^. Neurogenesis generally occurs in the subgranular zone (SGZ) of the hippocampal dentate gyrus^17^. Consequently, to assess neuroplasticity, we investigated the expression of SOX-2 and nestin in the SGZ of young adult vs. aged mice, as well as in PDD005-treated aged mice. SOX-2 expression was significantly decreased in aged mice vs. young adult mice (52.68% reduction, p = 0.011; Fig. 3A,B). However, the expression of SOX-2 in aged mice was restored to the level observed in young adult mice through PDD005-treatment. In the same manner, nestin expression was decreased in aged mice compared to young adults; however it was normalized in the SGZ of aged mice to a level comparable to that of young adults (P = 0.014 Fig. 3C,D) through PDD005-treatement. When compared to Donepezil, there was no statistical effect on SOX-2 or nestin expression in the SGZ of aged mice. Based on these results, it appears that PDD005 likely has a higher potential to rescue neuroplasticity decline, compared to Donepezil in the current study. When considering the potential involvement of hippocampal neurogenesis in the pathogenesis of NDs, these results suggest that PDD005-treatment promote neurogenesis, leading to improved cognitive performance in aging mice.

### PDD005 attenuates IL-1β synthesis and astrocyte and microglia activation in the subgranular zone of the aging brain

**Figure 4:**
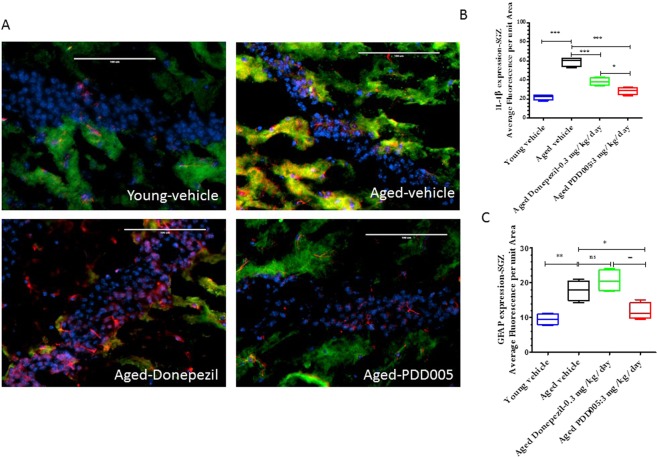
IL-1 β synthesis, astrocyte activation modulation in PDD005-treated aged mice. (**A**) Images showing IL-1β (green fluorescence), GFAP (red fluorescence) expression. Yellow = Overlay, blue = nuclei.- Nuclei; SGZ region of mouse brain.40X magnification, scale bar = 100 μm. **(B,C**) Graphs representing the average relative fluorescence of IL-1β (**B**), and GFAP in SGZ. Significant differences determined by using one-way ANOVA with Tukey’s test. *P < 0.050, **P < 0.01 and ***P < 0.001 compared with aged-vehicle.

**Figure 5:**
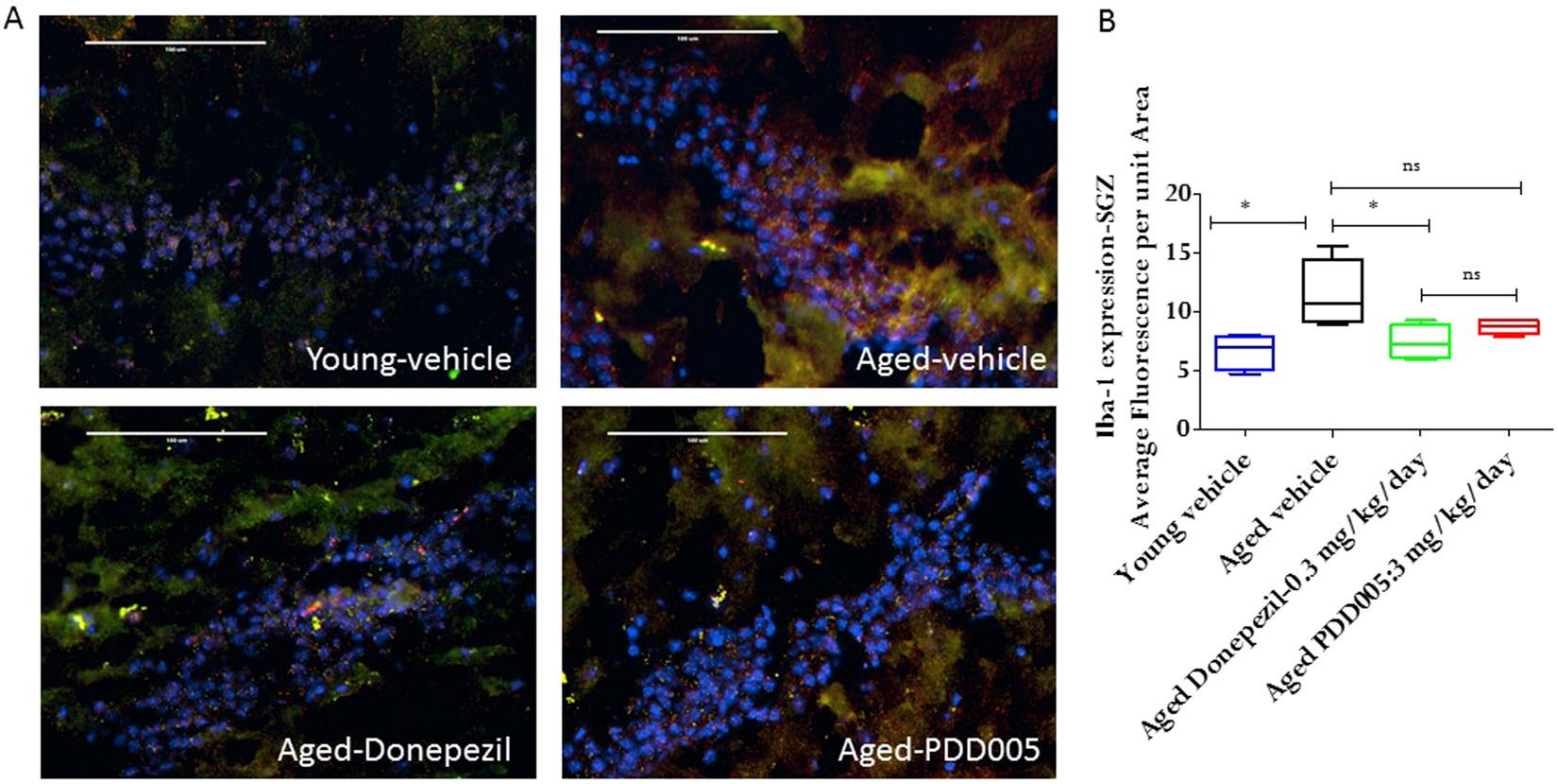
IL-1 β synthesis, and microglial activation modulation in PDD005-treated aged mice. (**A)** Images showing IL-1β (green fluorescence), iba-1 (red fluorescence) expression. Yellow = Overlay; Blue = nuclei; SGZ region of mouse brain. 40X magnification, scale bar = 100 μm. **(B**) Graphs representing the average relative fluorescence of Iba-1 in SGZ. Significant differences determined by using one-way ANOVA with Tukey’s test. *P < 0.050, **P < 0.01 and ***P < 0.001 compared with aged-vehicle.

It has been reported that markers of neuroinflammation, such as interleukin (IL)-1β, significantly increase in the brains of aging mice^16^. Thus, it is plausible that attenuation of the increased inflammatory response in the brain may alleviate cognitive deficits. We quantified IL-1β in SGZ from young and aged mice with or without PDD005-treatment. Notably, IL-1β expression increased significantly in aged mice, compared to young mice (2.7-fold, P < 0.0001; Fig. 4A,B). The observed increase in IL-1β synthesis was associated with a significant increase of glial fibrillary acidic protein (GFAP), a marker of astrocyte activation (P = 0.0002; Fig. 4A,C). We observed a significant reduction of IL-1β synthesis in the SGZ of PDD005-treated aged mice, compared to Donepezil-treated aged mice (Fig. 4A,B). Additionally, in contrast to Donepezil, PDD005-treatment also attenuated astrocyte activation in the SGZ of aged mice (P < 0.05; Fig. 4A,C). The microglia marker, ionized calcium-binding adaptor molecule 1 (Iba-1), was also observed to increase with age in the SGZ (Fig. 5A,B); however, PDD005-treatment did not significantly alter the observed microglial activation.

### PDD005 promotes prohibitin expression in aging mice

**Figure 6:**
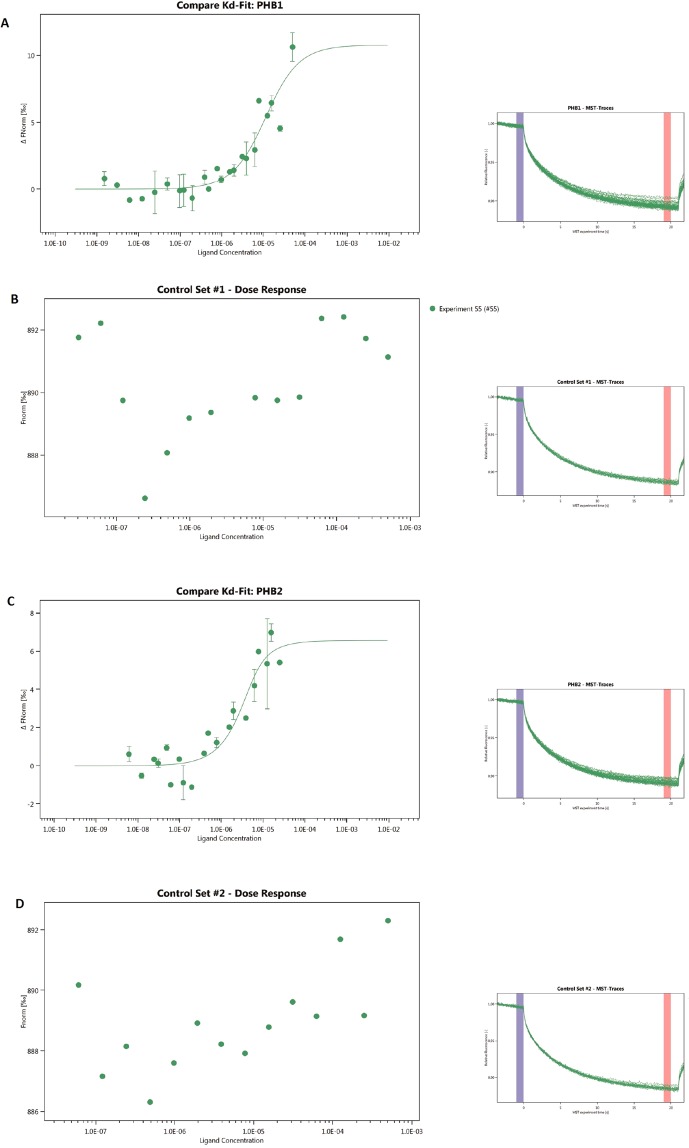
Prohibitin 1 and 2 as targets for PDD005. MST experiments showed that PDD005 interacts with PHB1 (**A**) and PHB2 (**C**) k_D_ of the interaction between PDD005 and PHB1 [9.50 × 10^−6^ ± 4.60 M (1.05 standard error of regression; 10.76 response amplitude and 10.72 signal to noise)] and of PDD005 with PHB2 [1.29 × 10^−6^ ± 1.16 M (0.84 standard error of regression; 6.56 response amplitude and 8.26 signal to noise)].Control experiments of PHB1 (**B**) or PHB2 (**D**) without PDD005.

Analogs of PDD005 have been reported to bind to PHBs^18^. PHBs are highly conserved proteins of the inner mitochondrial membrane, and are also found in the nucleus, endoplasmic reticulum, and plasma membrane^18,19^. PHBs are scaffold proteins that modulate many signaling pathways, including the nuclear factor-κ-light-chain-enhancer of activated B cells (NF-ĸB)^20^. In the mitochondria, PHBs stabilize mitochondrial proteins and modulate the mitochondrial complex to protect the cell from oxidative stress. We hypothesized that PDD005 binds to PHBs in lipid rafts, leading to their translocation into the mitochondria and subsequent activation of signaling pathways. Using microscale thermophoresis (MST) technology, the strength of the interactions between an intrinsically fluorescent sample and a ligand are measured while a temperature gradient is applied over time. If a binding event occurs, changes in MST signal are easily detected and binding affinity (k_D_) can be calculated from a fitted curve that plots normalized fluorescence against ligand concentration. MST experiments show that PDD005 interacts with PHB1 (Fig. 6A) and PHB2 (Fig. 6C) with a k_D_ of 9.50 × 10^−6^ ± 4.60 M (0.84 standard error of regression; 10.76 response amplitude and 10.72 signal to noise), and of 1.29 × 10^−6^ ± 1.16 M (0.84 standard error of regression; 6.56 response amplitude and 8.26 signal to noise), respectively. No interaction of MST buffer with PHB1 (Fig. 6B) or PHB2 (Fig. 6D) was observed.

**Figure 7:**
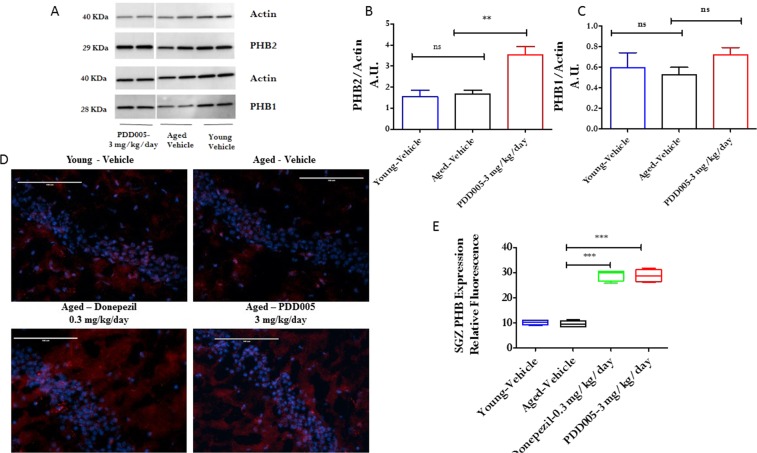
PDD005 enhances prohibitin expression in aged mice. (**A**) Representative western immunoblot for PHB1 and PHB2 in young adult-vehicle (n = 3), aged-vehicle (n = 4) and aged treated with PDD005 (SC, 3 mg/kg/day for 28 days, n = 4). (**B,C**) graphs showing analysis of PHB2 and PHB1 expression by western blot. (**D**) Images showing prohibitin (red) expression in SGZ; blue = nuclei. 40X magnification, scale bar = 100 μm. (**E**) Graph representing the average relative fluorescence of PHB. Data expressed as mean ± SEM of n = 4 mice per group. Significant differences determined by using one-way ANOVA with Tukey’s test. Ns = not significant; **P < 0.01 and ***P < 0.001 compared to the aged vehicle.

When considering the putative binding of PDD005 to PHB, we investigated the regulation of PHB expression by PDD005 in the aging brain. To do this, we quantified PHB1 and PHB2 by western blot in total brain extract and by immunofluorescence in the SGZ. The brain extracts from the *in vivo* experiments did not show a statistical reduction of either PHB1 or PHB2 in aged mice compared to young mice, as quantified by western blot (Fig. 7A,C). Interestingly, we found that chronic PDD005-treatment (SC, 3 mg/kg/day for 28 days) induced PHB1 and significantly increased the expression of PHB2 (*P* < 0.001) in aged mice. Immunofluorescent analysis confirmed that PDD005-treatment significantly induced PHB expression in the SGZ of the brain of aged mice (Fig. 7D,E). Taken together, our data demonstrate an association between IL-1β expression and PHB in the SGZ of the aged mouse brain.

### PDD005 reduces tau phosphorylation, IL-1β synthesis and promotes GSK-3β expression in 3-TgxAD mice

**Figure 8:**
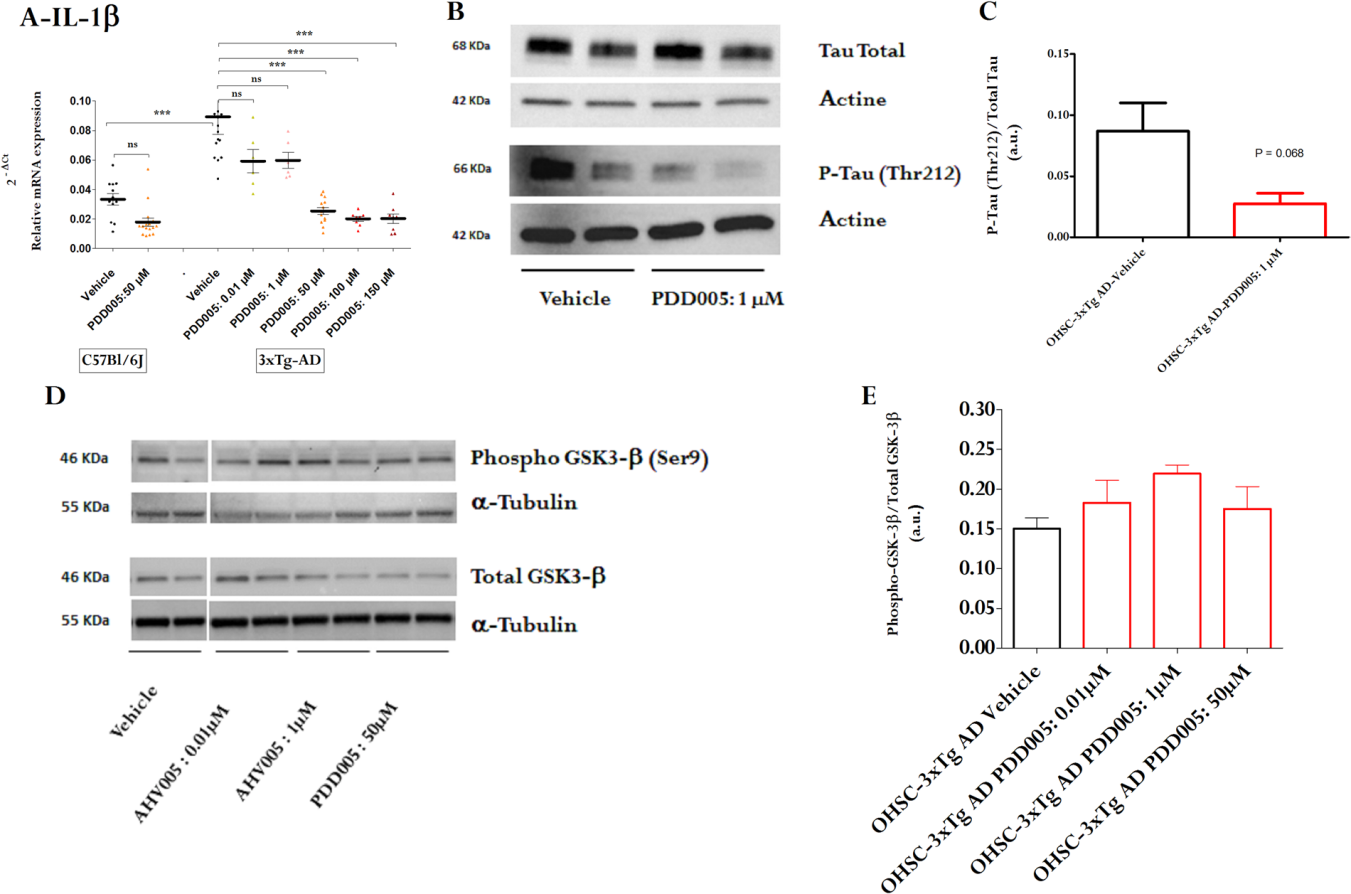
PDD005 modulates tau phosphorylation, IL-1β, GSK-3β in organotypic hippocampal slice cultures (OHSCs)-from 3x Tg- AD mice. (**A)** Graph illustrating the effect of PDD005-mediated attenuation of IL-1β in OHSCs from transgenic tau mice (3xTg-AD). Transcriptional expression of *IL-1β* was quantified by RT-PCR. Data expressed as the relative expression normalized to the housekeeping gene *HPRT* (2^−ΔCt^). Significant differences determined by using one-way ANOVA with Tukey’s test. *P < 0.05 and ***P < 0.001 indicate significant differences between PDD005 and the vehicle group. Data expressed as means ± SEM with n = 2–6 inserts/condition and 10 slices/insert. (**B,C**) Representative immunoblot of the effect of PDD005-mediated attenuation of tau phosphorylation (Thr 212) in OHSCs-3x Tg- AD mice and densitometric analysis of intensity of immunoblots. Data expressed as means ± SD with n = 2 inserts/condition and 10 slices/insert. Significant differences determined by using Student’s t test.*P < 0.05 indicates significant differences between PDD005 and the vehicle group. (**D,E**) Representative immunoblot of the effect of PDD005-mediated phosphorylation of GSK-3β in OHSCs in 3x Tg- AD mice (**D**) and densitometric analysis of intensity of immunoblots (**E**). Each bar is expressed as mean ± SEM (n = 4 mice for all groups). Significant differences determined by using one-way ANOVA with Tukey’s test. ns = not significant *P < 0.05 compared with vehicle group.

As attenuation of IL-1β signaling has been associated with a reduction of tau pathology, we investigated whether PDD005-treatment alters tau phosphorylation. PDD005-treatement reduced IL-1β expression in organotypic hippocampal slice cultures (OHSCs)-from 3x Tg- AD mice, overexpressing phosphorylated tau protein, as well as in WT mice (Fig. 8A). Interestingly, subchronic PDD005-treatment (10 days) attenuated tau phosphorylation in the OHSCs from 3x Tg-AD mice (Fig. 8B,C). This downregulation was associated with inhibition of GSK-3β activation (enhancement of GSK-3β phosphorylation) **(**Fig. 8D,E). Our observations suggest that the GSK-3β signaling pathway may be involved in the PDD005 mechanism of action. Altogether, our findings are in agreement with previous reports that suggest attenuating IL-1β signaling may offer therapeutic benefit to Alzheimer’s disease patients^21^, as well as provides a potential mechanism by which PDD005 downregulates IL-1β synthesis and tau protein phosphorylation.

## Discussion

**Figure 9:**
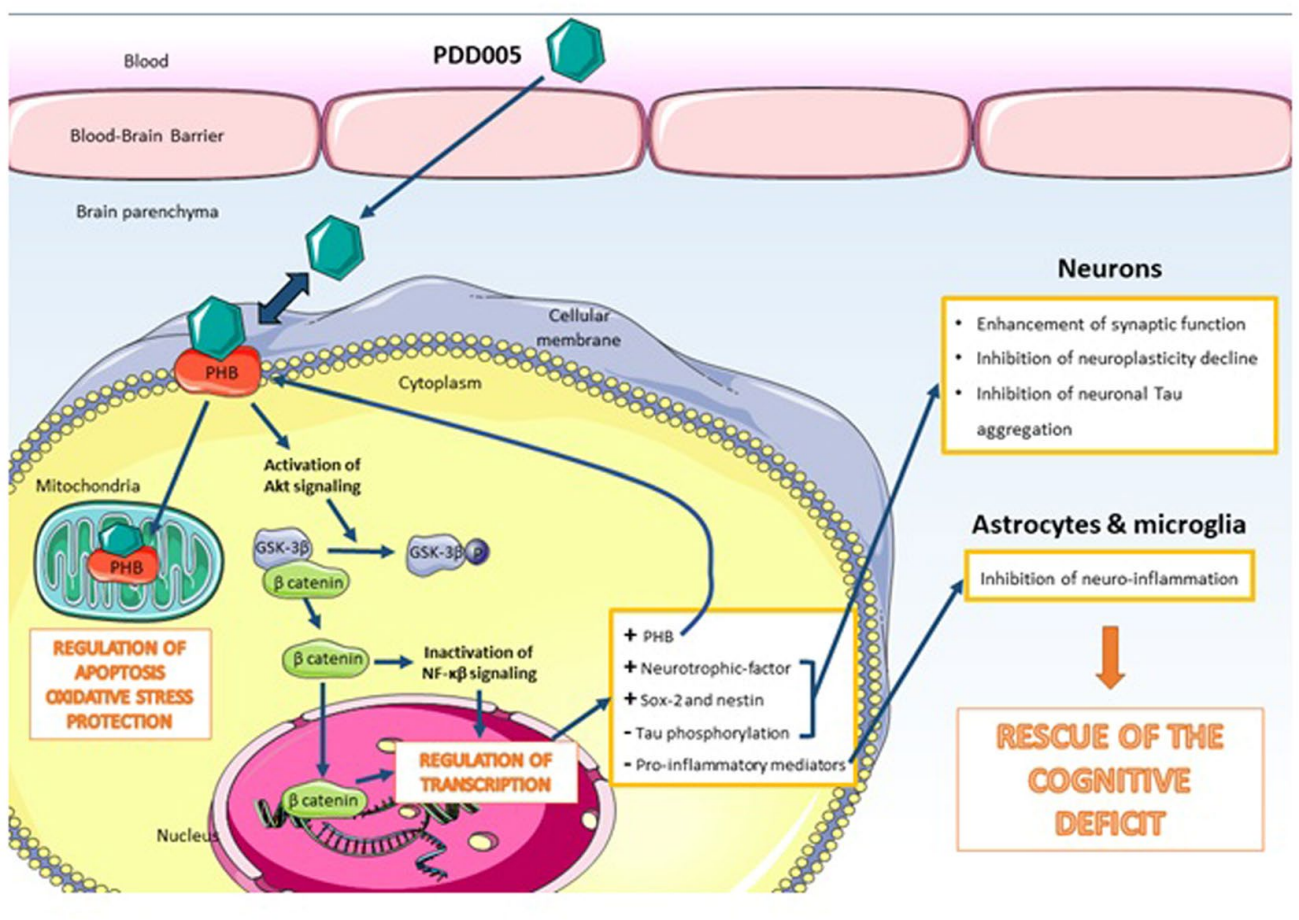
Main study findings and potential mechanisms of action of PDD005 in CNS cells.

We have identified a purine derivative drug (PDD005), which has a tertiary N6 amine position, shows promising therapeutic implications for the treatment of NDs. In the present study, we confirmed that PDD005 can be distributed to the brain. After crossing the BBB, our *in vivo* studies show that PDD005-treatment in aged mice is associated with: **(1)** a rescue of cognitive/memory deficit, **(2)** enhanced synaptic markers, **(3)** increased neurogenesis, **(4)** an attenuation of neuroinflammation and astrocyte activation, (**5**) increased PHB expression, and (**6**) activation of GSK-3β mediating β-catenin signaling pathways, which may mediate inhibition of neuronal tau hyperphosphorylation. Collectively, the results of this study suggest that PDD005-treatment works through multiple mechanisms of action to produce therapeutic effects NDs, such as Alzheimer’s disease, as summarized in Fig. 9.

NDs are associated with memory loss, including impairment of working memory, spatial memory, and recognition memory. Each of these mechanisms can be modulated through neurogenesis in the brain, which occurs throughout adulthood^22^. In the present study, we show that PDD005-treatment rescues cognitive/memory deficits *in vivo*. To investigate the potential of our new drug in the treatment of NDs and inflammatory disease, we chose to compare endpoints between wild-type young adult mice and aged mice. First, we demonstrated that spatial memory was improved after PDD005-treatment in young adult mice. Next, we showed that the cognitive decline observed with aging is attenuated with PDD005-treatment, as indicated by the results from the T-maze continuous alternation task. Taken together, these results suggest that PDD005-treatment can improve cognitive and memory performance, which is therapeutically beneficial in the treatment of NDs.

There are multiple mechanisms involved in the pathophysiology of NDs, and many are closely related. Such mechanisms include altered synaptic function and neuroplasticity, abnormal neuroimmune response, and misfolded/aggregated protein accumulation leading to amyloid plaques and neurofibrillary tangles. As the molecular pathways involved in the pathogenesis of many different NDs are connected, and the timing of their activity in the pathogenesis of the disease is still unknown, there is an immediate need to develop pharmaceuticals that have multiple mechanisms of action involving key regulatory pathways. Thus, we investigated the effect of PDD005 on neuronal abnormality, neuroinflammation, and tau hyperphosphorylation in the aging brain. Abnormalities in the expression of immediate early genes that play a role in critical memory formation have been reported in the brain in several NDs. Among these genes, synaptophysin (*SYP*) and postsynaptic density protein (*PSD95*) can be used to assess synaptic function. Our results show that aged mice display decreased *SYP* and *PSD95* transcriptional expression in the brain, compared to young adult mice, which can be normalized through PDD005-treatment. Collectively, our results suggest that the improvement of cognitive and memory performance observed with PDD005-treatment in our aged mice is related to enhanced synaptic function.

Synaptic plasticity is important for memory processing^23^. Evidence of impaired synaptic plasticity in NDs provides further insight into the association between neurodegeneration and memory deficit. For example, SOX-2 expression is decreased in the brain of the transgenic Alzheimer’s disease mouse model^16,24–26^, as well as in the brains of Alzheimer’s disease patients^24^. Interestingly, neurogenic niches also show increased hyperphosphorylated tau protein and neurogenic impairment has been found to precede the onset of amyloid deposition and memory deficits in a rodent model^16^. In Alzheimer’s patients, impairment of synaptic plasticity correlates with the severity of cognitive decline^27^; in mouse models, the decline in neurogenesis is associated with cognitive impairment during aging^28^. The number of SOX-2- and nestin-expressing cells is also reported to be reduced in the dentate gyrus of Parkinson’s disease patients with dementia^7,9^. Our results show that PDD005-treatment results in enhanced SOX-2 and nestin expression in the SGZ of aged mice, suggesting that PDD005 may promote synaptic plasticity. As such, PDD005 shows promise in normalizing neuronal function through two distinct mechanisms: 1) enhancement of synaptic markers, and 2) inhibition of the decline in neuroplasticity observed with aging.

Increasing evidence suggests that the pathogenesis of NDs is not restricted to neuronal alterations, but also includes inflammatory mechanisms in the brain^29,30^. For example, aggregated proteins observed in the brain of patients with Alzheimer’s disease are known to bind to pattern recognition receptors, on the microglia and astroglia, and trigger release of inflammatory mediators that contribute to disease progression and severity^31^. For example, chronic inflammatory stimuli have been shown to inhibit the protective and regenerative ability of neuronal stem cells^32–34^. As such, targeting neuroimmune mechanisms in the aging brain is another potential strategy to treat NDs effectively. In agreement with the literature, our data show increased IL-1β expression, and activation of astroglia and microglia, in the brains of aged mice compared to young mice, which was attenuated with PDD005-treatment. Neurogenic niches show an increase of hyperphosphorylated tau protein^16^ and neurogenic impairments have been found to precede the onset of amyloid deposition and memory deficit in a rodent model. We explored the potential beneficial effect of PDD005-treatment on this processes, associated with progression of Alzheimer’s disease. To do this, we used organotypic hippocampal slice cultures (OHSCs) from a rodent Alzheimer’s disease model, 3x Tg-AD mice. In this *ex vivo* model, we demonstrated that PDD005-treatment attenuates tau hyperphosphorylation. Consistent with *in vivo* observations in the aged brain, this effect was associated with decreased IL-1β synthesis. These findings are in agreement with previous observations showing a link between IL-1β expression, tau hyperphosphorylation, and neurodegeneration in the brain^35^.

Emerging evidence suggests that PHB1 and PHB2 play a role in Parkinson’s and Alzheimer’s diseases^36^. PHB is reported in the literature to be a key regulator of neuronal survival and is also suggested to be involved in neuroplasticity^37^. Neuronal-specific depletion of PHB2 results in an aberrant mitochondrial ultrastructure leading to behavioral impairment and cognitive deficiencies^36^. In the current study, we demonstrated that PDD005 interacts with PHB. Surprisingly, we observed no significant decrease of PHB1 and PHB2 expression in the brains of aged mice. However, there was an increase in both PHB1 and PHB2 expression in the brains of PDD005-treated aged mice, which correlated with decreased expression of IL-1β in the SGZ. This *in vivo* result further supports our hypothesis that PDD005 might interact with PHB to produce both neuroprotective and anti-inflammatory effects.

Our findings suggest that GSK-3β may be involved in mediating the neuroprotective effects of PDD005 in the brain. GSK-3β is a signaling molecule that plays a central role in a diverse range of signaling pathways. Among others, GSK-3-mediated phosphorylation triggers β-catenin destabilization and modulation of gene transcription^38^. Released β-catenin can also interact with components of NF-kβ to inhibit transcription of target genes, including the transcription of proinflammatory mediators^39^. Our results suggest that GSK-3 is one of the mediators in the downstream signaling pathway of PHB. This signal transduction pathway may result in PDD005-mediated gene expression, including modulation of neurotrophic factors and synaptic molecule expression in neurons. Other mediators and signaling pathways involved, such as β-catenin and NF-kβ, have yet to be studied.

The current study provides evidence that PDD005 is able to translocate across the BBB, rescue cognitive deficit, attenuate neuro-inflammation, reduce the decline in neurogenesis, and restore synaptic function in aged mice. These findings were associated with an increase in PHB in the SGZ of PDD005-treated aged mice. The significant increase of PHB mediated by PDD005-treatment in the aged mice was also observed in organotypic hippocampal slices (data not shown) associated with the reduction of phospho-tau. The findings from this preclinical study in aged mice, as well as in the OHSCs-3xTg-AD *ex vivo* model, provide evidence that regulation of PHB may be one of the mechanistic pathways involved in the rescue of a neurocognitive deficit, neuroplasticity repair, IL-1β synthesis regulation, and neuroprotection by PDD005. This notion is further supported by our results which demonstrate the interaction of PDD005 with PHB1 and PHB2. Our data suggest that both targets may be involved in the therapeutic mechanism of action of PDD005 in the brains of aged mice. Although additional studies are required to further demonstrate the exact intracellular mechanisms involved, this proof-of-concept preclinical study highlights the potential interest of a purine derivative, with a tertiary N6 amine position, for cognitive deficit rescue in aged mice and treatment of tau pathology.

## Materials and Methods

### Chemical synthesis

Reactions were monitored by TLC using Merck silica gel 60F-254 thin-layer plates. Column chromatography (CC) experiments were performed using silica gel (70–200 µm). Melting points were determined on a Kofler hot stage (Reichert) and are uncorrected. NMR spectra were recorded on Bruker Avance 400 MHz. The HPLC analyses were carried out on a system consisting of a Waters 600 system.

*Synthesis of PDD005*: (2 R)-2-[Amino-[6-(benzyl(methyl)amino)-9-isopropyl-purin-2-yl]amino]butan-1-ol (**4**).

**Scheme 1:**
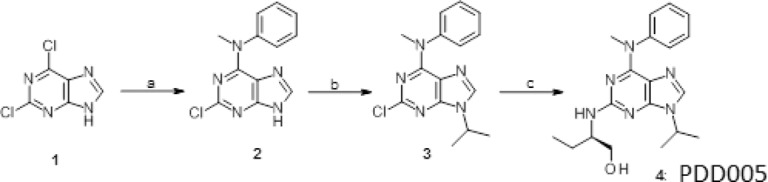
Reagents and conditions. (**a**) N-methylaniline, N (Et)_3_, in n-butanol, 95 °C; (**b**) *iso*-propylbromide, K_2_CO_3_, DMSO 16–18 °C; (**c**) (R)-2-aminobutanol, 160 °C.

The preparation of PDD005 (**4**) is depicted in Scheme 1.

N-methylaniline 8.6 mL 79.36 mmol and NEt_3_ (29 mL, 211.63 mmol) were added to a solution of 2,6-dichloropurine (10.0 g, 52.91 mmol) in *n*-butanol (80 mL). The mixture was heated at 100 °C for 3 h. The reaction was cooled to 40 °C, the precipitate of compound #2 was filtered as this temperature, washed with 10 mL of cold (15 °C) water and dried overnight under vacuum. Compound #**2** was isolated in 81% yield and used for the next step. In the second step, 2-bromopropane 10.22 g, 39.34 mmol) and K_2_CO_3_ (21.74 g, 157.35 mmol) in 80 mL DMSO. After 12 h of stirring, the mixture was poured in 200 mL of water and extracted with AcOEt (3 × 20 mL). Compound #**3**, which crystallized upon concentration of the organic layer, was triturated with AcOEt (5 mL) and dried. A mixture of the 2-chloropurine **3** and of (R)-2-aminobutanol was heated 4 hours at 160 °C. After cooling, the mixture was extracted with CH_2_Cl_2_, washed with water (2 × 20 mL). Derivative compound #3 crystallized after the evaporation of the solvent upon trituration with AcOEt. NMR spectra were recorded and are shown in Fig. S1.

### *In vitro* study

#### Isolation of rat brain endothelial and glial cells

Rat primary brain endothelial cells (BECs) and primary glial cells were isolated and cultured as previously described^15^. Briefly, for BECs, brain tissues was digested enzymatically (1 g/L collagenase/dispase, 20 U/mL DNAse I, 0.147 mg/L *N*_α_-tosyl-L-lysine chloromethyl ketone hydrochloride in HBSS medium, 1 h at 37 °C). All reagents were from Sigma-Aldrich (Saint-Quentin Fallavier, France) except HBSS medium, which was from Gibco (Fisher Scientific, Illkirch, France). A 20% BSA (Sigma-Aldrich, Saint-Quentin Fallavier) gradient was used for isolation of capillaries. After a second enzymatic digestion, cells were plated in 75 cm^2^ coated culture flasks in endothelial cell basal medium-2 completed by the EGM-2 MV SingleQuots kit (LONZA, Basel, Switzerland). Cultures were maintained at 37 °C in a humidified 5% CO_2_ atmosphere for 5–6 days before being trypsinized and frozen. For rat primary glial cells, brain tissue from two-day old pups were digested for 30 min by 0.025% trypsin (Fisher Scientific, Illkirch, France) after removal of meninges, olfactory bulbs, and cerebellum. Then, homogenates were filtered (40 µm pore) and plated in 75 cm^2^ coated (poly-L-lysine solution 0.0001%, Sigma-Aldrich, Saint-Quentin Fallavier, France) culture flasks in glial specific medium (mix of α-MEM and F12 medium, Fisher Scientific, Illkirch, France) supplemented with 5 ng/mL basic FGF protein (Millipore, Fontenay-sous-Bois, France), 1% human serum (Sigma-Aldrich, Saint-Quentin Fallavier, France), 5% fetal bovine serum and 1% penicillin-streptomycin-neomycin antibiotic mixture (PSN) (Fisher Scientific, Illkirch, France). Cultures were maintained at 37 °C in a humidified 5% CO_2_ atmosphere for 5–6 days before being trypsinized and frozen.

#### *In vitro* cell-based rat blood-brain barrier (BBB) model

*In vitro* cell-based rat BBB model was set-up as we described previously^15^. Briefly, Glial cells (2 × 10^4^ cells) were seeded on plates in the glial specific medium. After 24 h, BECs (6 × 10^4^ cells) were plated on the upper side of a polycarbonate Tanswell membrane (pore size 0.4 µm; diameter 12 mm; insert growth area 1.12 cm^2^; Corning, Sigma-Aldrich, Saint-Quentin Fallavier, France) in 0.5 mL of the endothelial cell basal medium-2 (LONZA, Basel, Switzerland) containing 0.1% human epidermal growth factor (hEGF), 0.04% hydrocortisone, 0.1% R3-insulin-like growth factor-1, 0.1% ascorbic acid, 0.4% human fibroblast growth factor-β, 0.1% mix of gentamicin/amphotericin-B-1000 and 5% fetal bovine serum (LONZA, Basel, Switzerland). The chambers containing glial cells and BECs were considered as the basolateral and apical compartment, respectively. The plates were then incubated at 37 °C in a 5% CO_2_ atmosphere. Under these experimental conditions, BECs formed a confluent monolayer within 7 to 10 days as we previously reported^15^. The integrity of the cell-based BBB models was demonstrated as we reported previously^15^ by (1) assessing the presence of tight junctions between the BECs (2) measuring the flux of the paracellular reference marker, [^14^C]-sucrose (Perkin Elmer, Courtaboeuf, France) through the monolayer, and (3) by determining the functional polarity of BECs by the assessment of P-glycoprotein (P-gp) substrate vinblastine efflux ([^3^H]-vinblastine sulphate, Perkin Elmer, Courtaboeuf, France).

#### Analysis of PDD005 translocation across the cell-based BBB model

After checking BEC monolayer integrity, TRANSWELLS were transferred to new plates as we described previously^15^. 1.5 mL of T buffer (150 mM NaCl, 5.2 mM KCl, 2.2 mM CaCl_2_, 0.2 mM MgCl_2_, 6 mM NaHCO_3_, 3 mM glucose and 5 mM Hepes, all from Sigma-Aldrich, Saint-Quentin Fallavier, France) was added to the basal chamber (B) and 0.5 mL to the apical chamber (A). Experiments were performed in triplicate. PDD005 (10 µM) was introduced into the the apical or tbasal compartment as we reported previously^15^. After 60 min, aliquots were removed from the apical or basal chambers for drug concentration determination by liquid chromatography coupled to mass spectrometry as we described previously^15^. The apparent permeability (P_app_) value was calculated as we described previously^15^ and as follows: P_app_ = dQ/dT x A x C_0_ where dQ/dT: amount of drug transported per time-point; A: membrane surface area; C_0_: donor concentration at time-point 0. Data are presented as the average mean ± SD from three monolayers. Mass balance of all compounds was between 80% and 120%. The mass balance^15^ was calculated as follows: R (%) = [(Ap + Bs)/A_0_] × 100 where Ap and Bs are the amount of tested compounds in the apical and basal compartments, respectively, and A_0_ is the initial amount in the donor compartment.

#### Organotypic hippocampal slice cultures

Organotypic hippocampal slice cultures (OHSCs) were prepared from newborn (postnatal day 5 to 8) 3xTg AD mice (kindly provided by Dr Michel De Chaldée, CEA Saclay, I2BC) according the interface method^40^, with minor modifications. Briefly, hippocampi were dissected in a dissection medium (MEM [Fisher Scientific, Illkirch, France] with 33 mM final glucose [Sigma-Aldrich, Saint-Quentin Fallavier, France]) and included in 2% agarose block. The OHSCs of 400 µm were obtained with a microtome with vibrating blade (Microm Microtech, France) and agarose was removed from the slices. OHSCs were seeded on 6-well dry inserts and kept for 7 days *in vitro* at 37 °C in a humidified atmosphere (5% CO_2_) with 1.1 mL feeding medium in basolateral compartment (25% HBSS medium, 49% opti-MEM medium, 25% heat-inactivated horse serum and 1% of PSN, pH 7.4; all reagents from Gibco, Fisher Scientific, Illkirch, France). Feeding medium was half-changed at DIV1 and every two or three days. At DIV7, feeding medium was completely removed and replaced by treatment medium (25% HBSS medium, 49% opti-MEM medium, 14% Neurobasal-A medium, 1% B-27 Serum-Free supplement, 10% heat-inactivated horse serum and 1% of PSN, pH 7.4, all from Fisher Scientific, Illkirch, France) with the different concentrations of PDD005 for 11 days. Half of the volume of treatment medium was changed every 2–3 days. At DIV18, the cultures were stopped by washing slices with cold HBSS medium containing 4% cOmplete Protease Inhibitor Cocktail (Sigma-Aldrich, Saint-Quentin Fallavier, France) and 20% of a mix of anti-phosphatase inhibitors (0.5 mM ammonium molybdate tetrahydrate, 0.1 M β-glycerophosphate disodium salt hydrate, 0.25 M sodium fluoride, 50 mM sodium pyrophosphate decahydrate and 5 mM sodium orthovanadate; all reagents from Sigma-Aldrich, Saint-Quentin Fallavier, France). Then, some OHSCs were collected in microtubes, centrifuged and frozen for gene expression studies. The other OHSCs were treated in freshly prepared lysis buffer containing 50 mM Tris-HCl pH 8.5, 2 mM EDTA, 2 mM EGTA, 100 mM sodium chloride, 0.2% SDS 10% and 1% TRITON X-100 10% (all reagents from Sigma-Aldrich, Saint-Quentin Fallavier, France) supplemented with 4% of Complete Protease Inhibitor Cocktail and 20% of a mix of anti-phosphatase inhibitors. After homogenization, OHSCs were centrifuged at 16 000 × g for 15 minutes. Supernatants were recovered and frozen for further protein experiments.

### *In vivo* study

#### Animals

Male 2- to 3-month-old C57Bl/6 J (young mice) and 12-month-old C57Bl/6 J mice (aged mice) were used. Animals were kept under monitored specific and opportunistic pathogen-free conditions. This study was conducted with the approval by the Institutional Animal Care and Use Committees (National Ethic committee for animal experiments n° CEEA35, France) and was carried out in compliance with European legislation on animal care and scientific experimentation.

For pharmacokinetic studies, a single intraperitoneal injection (i.p.) (10 mg/kg), single oral administration (PO) (5 mg/kg), or chronic subcutaneous injection (SC) (28 days, 8 mg/kg/day) using osmotic minipumps (ALZET model 2004, Q = 0.25 μL/h DURECT, Cupertino, CA, USA) of PDD005 was performed, while a subchronic study of repeated PDD005 i.p. (28 days, 3 mg/kg/day) was used for cognitive function studies in aging mice.

### Behavior studies

#### Y-maze

This study was conducted with the approval by the Institutional Animal Care and Use Committees (ComETH, ILkirch, France) and was carried out in compliance with European legislation on animal care and scientific experimentation. 2- to 3-month-old C57Bl/6 mice were used for cognitive performance studies. The mice were randomly distributed to different experimental groups (13–15 animals per group). Mice were then exposed by SC injection to PDD005 at 8 mg/kg/day or vehicle for 28 days. The spontaneous alternation test (Y maze) evaluates the short-term memory (working memory) of the animal. This test is carried out in a Y-maze with arms customized by different shapes; squares (A), triangles (B) and lines (C). For this test, a mouse is placed in one of the arms for 8 min and is free to explore spontaneously through the three arms of the Y-maze. During the session, the number of entries of each arm of the Y-maze is counted. At the end of the session, the number of possibilities (number of total entries in the arms) and the number of spontaneous alternations are calculated. An alternation is defined by consecutive entries in the three different arms.

$${\rm{Alternation}}\,( \% )=\frac{{\rm{Nb}}\,{\rm{of}}\,{\rm{alternations}}}{{\rm{Total}}\,{\rm{nb}}\,{\rm{of}}\,{\rm{arm}}\,{\rm{entries}}\,{\rm{minus}}\,1}\times 100$$

A mouse with a normal phenotype will tend to explore the novelty. Thus, the percentage alternation will be higher in a mouse without impaired working memory.

#### T-Maze

12-month-old C57Bl/6 mice were used as aged specimens. 2- to 3-month-old mice (young adults) were used as animals with an intact level of cognitive/memory performance. The mice were randomly distributed to different experimental groups (10 animals per group). PDD005 was administrated i.p. at 3 mg/kg/day for 28 days. The T-maze session was carried out as we described previously^41^. The T-maze apparatus consisted of two choice arms (goal arms) interconnected at 90° to a main stem that comprises a start box. Sliding doors were used to briefly restrain the mouse in the start box at the initiation of the test or to close a specific goal arms during the forced choice alternation task. The T-maze session was composed of an initial “forced-choice” trial followed by 14 “free-choice” trials. The forced choice trial began by the release of the animal from the start box while one goal arm was closed. Once the animal entered the open goal arm, the forced choice trial is considered completed. After the return of the animal to the start arm, the left and right goal door were opened so that the animal could choose freely between the left or right goal arm (free choice trial). The T-maze session is considered completed when 14 free-choice trials have been performed or 15 min have elapsed, whichever occurs first. The percent spontaneous alternations was calculated as the number of spontaneous alternations divided by the number of free-choice trials”.

### LC-MS/MS analysis

Liquid chromatography tandem mass chromatography LC-MS/MS technic was used to determine PDD005 concentration in the media from the *in vitro* study and in both plasma and brain homogenates from the *in vivo* study as we described previously^15,42^. Mobile phase A was H_2_O + 0.1% HCOOH and mobile phase B was ACN + 0.1% HCOOH. Brains were mixed in a buffer containing 4% of complete Protease Inhibitor cocktail and phosphatase inhibitors using a Precellys24 tissue homogenizer with soft tissue homogenizing tubes CK14 (Bertin Technologies, Montigny-le-Bretonneux, France). Brain homogenates and plasma (100 µL) were subjected to protein precipitation with 10 µL of methanol previously spiked with an internal standard (PDD 004–4 50 nM). The samples were then diluted 1:10 in ultrapure water and centrifuged at 25 000 g for 10 min at 4 °C. The supernatant was collected and used for solid-liquid extraction on Oasis HLB cc/30 mg cartridges (WAT094225, Waters, Saint-Quentin-en-Yvelines, France). Eluates were dried under nitrogen at 40 °C for about 20 min and then suspended in 100 µL of phase A/B 50/50. After centrifugation at 25 000 g at 4 °C for 10 min, the supernatants were collected for chromatography.

A Quattro Premier (Waters, Saint-Quentin-en-Yvelines, France) system on a UPLC column (Acquity UPLC BEH C18, #186002350, Waters) was coupled with a pre-column (Acquity UPLC BEH Shield RP18 VanGuard, #186003977, Waters). The total run time and the flow rate was 2.21 and 0.4 mL/min, respectively. Tandem mass spectrometry was used for the detection of analyte in a positive electrospray mode. As tuning parameters, capillary voltage and source temperature was 2.7 kV and 120 °C, respectively. The reaction monitoring transitions for the analyte was 355.36 m/z. Drug (PDD005) concentration in the brain and plasma extracts was determined using the calibration curves (ranges were from 1 nM to 300 nM) and PDD004 as internal standard. Xcalibur 2.2 software (Thermo Fisher Scientific, Bremen, Germany) was used for instrument control and processing of the data files. Partition coefficient (Kp, brain/plasma) was calculated as the ratio between brain (subtracting 2% of blood contamination) and plasma drug concentrations.

### Gene expression assessment by total RNA extraction and reverse transcription-quantitative PCR (RT-qPCR)

The effect of PDD005 on gene expression of different biomarkers was determined by measuring mRNA levels. S*ynaptophysin* (*SYP*) and *postsynaptic density protein 95* (P*SD95*) genes were evaluated in total brain from mice exposed to PDD005 at 3 mg/kg/day and *interleukin-1β* (*IL-1β*) gene from OHSCs-3xTg AD mice. Total RNA was extracted with QIAZOLreagent and purified on RNEASY Plus Universal Tissue Mini Kit columns (Qiagen, Courtaboeuf, France). Briefly, OHSCs were homogenized with 1 mL of QIAZOL reagent while brain tissue (30 mg) was homogenized with 1 mL of QIAZOL reagent using a Precellys24 tissue homogenizer. A treatment for elimination of gDNA was performed. After addition of 180 μL of chloroform, mixtures were centrifuged at 12 000 g for 15 min at 4 °C and aqueous phases were mixed with 600 μL of 70% ethanol and loaded onto RNEASY columns. Total RNA was washed and eluted with RNase-free water according to the manufacturer’s protocol and stored at −80 °C. RNA concentration was measured spectrophotometrically at 260 nm in a NanoDrop 2000c (Labtech France, Palaiseau, France) (sample A260 nm/A280 nm ratio about 2 indicated lack of protein and phenol contamination). cDNAs were synthesized from 0.5 μg of total RNA with the RT² HT First Strand kit (Qiagen, Courtaboeuf, France) according to the manufacturer’s protocol and stored at −80 °C. For qPCR, 2 μL of cDNA was mixed with 6.25 μL of iTaq Universal SYBR Green Supermix (Bio-Rad, Marnes-la-Coquette, France), 0.375 µL of 10 µM primer mix (see list below) and completed to 10 μL with ultra-pure DNase-RNase-free distilled water. The qPCR reactions were performed in a CFX96 Real-Time Detection System (Bio-Rad, Marnes-la-Coquette, France) with the following cycle conditions: denaturation at 95 °C for 10 min, 40 cycles of 15 sec at 95 °C followed by 1 min at 60 °C and finished by 30 sec at 72 °C. Threshold cycles (Ct) of target gene and housekeeping gene (hypoxanthine guanine phosphoribosyl transferase, *HPRT*) were recorded and gene expression was calculated as 2^−ΔCt^ (where ΔCt = Ct target − Ct *HPRT*). Specificity of PCR reactions were confirmed by melt curve analysis. The primer sequences used for amplification were from Sigma and are provided in Table S1.

### Immunoblotting

OHSC lysate or total brain lysate was used for the western blot assay. Briefly, brain tissue was homogenized with a Precellys24 tissue homogenizer in freshly prepared lysis buffer containing 20 mM Trizma-Base, 150 mM NaCl, pH 7.4 (Sigma-Aldrich, Saint-Quentin Fallavier, France) and supplemented with 1% Triton X-100, 4% of cOmplete Protease Inhibitor Cocktail and 20% of a mix of anti-phosphatase inhibitors. TSamples were then centrifuged at, both 2500 ×g (15 min) and 10 000 xg (20 min) on homogenates to produce lysate for electrophoresis. Proteins (10 µg) and protein standard in Laemmli buffer were loaded on 4%-15% CRITERION TGX STAIN-FREE protein gel in running buffer TGS 1 × (all from Bio-Rad, Marnes-la-Coquette, France) and transferred to AMERSHAM HYBOND P 0.45 µm PVDF membrane (VWR International, Fontenay-sous-Bois, France). Membranes were blocked for 30 minutes in 5% low-fat milk in TBS-Tween 20 0.1% at room temperature. Blots were incubate with primary antibodies (Table S2) overnight at 4 °C and detected by horseradish peroxidase secondary antibodies diluted 1:5 000 in 5% low-fat milk in TBS-Tween 20 0.1% at room temperature. Actin antibody was diluted in 5% nonfat dry milk in TBS-Tween 20 0.1% for protein expression normalization. For protein detection, membranes were exposed to CLARITY Western ECL substrate in a CHEMIDOC TOUC IMAGING SYSTEM for a measurable exposure time (Bio-Rad, Marnes-la-Coquette, France) and quantified with Image Lab Software (Bio-Rad, Marnes-la-Coquette, France).

### Protein expression assessment by immunofluorescence

Frozen brain sections (10 µm) of the cerebrum (between Bregma 0 through -2.5 mm) were prepared for either single endpoint or double immunofluorescence, using techniques previously described by our laboratory^43^ with the following primary antibodies: IL-1β (1:1000; Abcam, Cambridge, MA; Cat. #ab2105), nestin (1:500; Abcam #ab105389), SOX-2 (1:500; Abcam #ab171380), GFAP (1:500; Abcam #ab7260), Iba-1 (1:500; Novus #NB100–2833), prohibitin (1:500; Novus #NBP2–67334) and anti-rabbit Alexa Fluor 477 (1:250), anti-rabbit Alexa Fluor 555 (1:250), anti-mouse Alexa Fluor 555 (1:250) or anti-goat Alexa Flour 555 and/or anti-sheep Alexa Fluor 455 (1:500) secondary antibodies. Hoechst stain (1:10,000 dilution) was added to secondary antibodies to visualize nuclei. Each endpoint was processed and analyzed as a single batch. Slides (SGZ region) were imaged by fluorescence microscopy at 40x with the appropriate excitation/emission filter, digitally recorded, and analyzed using image densitometry with ImageJ software (NIH) by a blinded technician. Slides with no primary antibody were used as negative controls (data not shown). A minimum of 3–5 locations on each section (2 sections per slide), 3 slides and n = 4–5 per group were used for analysis, as previously described by our laboratory^43^.

### Microscale thermophoresis

The PHB-fluorescence proteins were adjusted to 8 μM with MST buffer supplemented with 0.05% Tween 20 (NanoTemper Technologies). PDD005 was dissolved in MST buffer supplemented with 0.05% Tween 20 and a series of 16 1:1 dilutions (ligand concentrations ranging from 200 µM to 6 μM) were prepared using the same buffer. For the measurement, each ligand dilution was mixed with one volume of PHB-fluorescence proteins (1 and 2 isoforms), leading to a final concentration of PHB-fluorescence proteins of 4 μM and final ligand concentrations ranging from 100 µM to 3 μM. After 10 min incubation followed by centrifugation at 10 000 × g for 10 min, the samples were loaded into standard Monolith NT.115 Capillaries and MST was measured using a Monolith NT.115 instrument (NanoTemper Technologies) at a temperature of 22 °C as described previously^44^. We adjusted the instrument parameters to 100% LED power and medium MST power (40%). We analyzed data from two independently measuremnts (MO.Affinity Analysis software version 2.3, NanoTemper Technologies) using the signal from an MST-on time of 20 s.

### Statistical analysis

All statistical analyses were performed out using GraphPad Prism software (Version 7.0). Experimental comparisons with multiple groups were analyzed using one-way ANOVA with Tukey’s multiple comparison test for post hoc analysis. For comparison of two groups, Mann-Whitney tests were performed and are noted in the figure legends. Spearman tests were performed for correlation studies between two parameters. A P value of 0.05 or less was considered significant.

## Supplementary information

Supplementary Information.
