# Author Correction: A Small Compound Targeting Prohibitin with Potential Interest for Cognitive Deficit Rescue in Aging mice and Tau Pathology Treatment

**DOI:** 10.1038/s41598-020-67127-x

**Published:** 2020-07-03

**Authors:** Anne-Cécile Guyot, Charlotte Leuxe, Clémence Disdier, Nassima Oumata, Narciso Costa, Gwenaëlle Le Roux, Paloma F. Varela, Arnaud Duchon, Jean Baptiste Charbonnier, Yann Herault, Serena Pavoni, Hervé Galons, Emile Andriambeloson, Stéphanie Wagner, Laurent Meijer, Amie K. Lund, Aloïse Mabondzo

**Affiliations:** 1 Service de Pharmacologie et d’Immunoanalyse, CEA, Université Paris-Saclay, F-91191 Gif-sur Yvette, France; 2 Department of Pediatrics, Women & Infants Hospital of Rhode Island, The Warren Alpert Medical School, Brown University, Providence, RI USA; 3 University Paris Descartes, INSERM U1022, 4, avenue de l’Observatoire, 75006 Paris, France; 4 Institute for Integrative Biology of the Cell: Department of Biochemistry, Biophysics and Structural Biology, CEA, Université Paris-Saclay, F-91191 Gif-Sur Yvette, France; 5 Université de Strasbourg, CNRS, INSERM, IGBMC, 1 rue Laurent Fries, 67404 Illkirch, France; 6 Service d’Etude des Prions et Infections Atypiques (SEPIA), Institut François Jacob, CEA, Université Paris-Saclay, Fontenay-aux-Roses, France; 7 Neurofit, 67400 Illkirch, France; 8 Perha-pharma, Centre de Perharidy, Roscoff, France; 9 Department of Biological Sciences, Advanced Environmental Research Institute, University of North Texas, Denton, TX USA

Correction to: *Scientific Reports* 10.1038/s41598-020-57560-3, published online 24 January 2020

The original version of this Article contained a typographical error in the spelling of the author Paloma F. Varela, which was incorrectly given as Paloma Fernandez-Varela. This has now been corrected in the PDF and HTML versions of the Article, and in the accompanying Supplementary Information file.

