# Supplementary material for: A Small Compound Targeting Prohibitin with Potential Interest for Cognitive Deficit Rescue in Aging mice and Tau Pathology Treatment
Source: Sci Rep. 2020 Jan 24;10:1143. doi: 10.1038/s41598-020-57560-3 (PMC6981120; doi:10.1038/s41598-020-57560-3)
Supplement: Supplementary file 1 — Supplementary Information. [file 41598_2020_57560_MOESM1_ESM.pdf]

Supplementary Materials

A Small Compound Targeting Prohibitin with Potential Interest for Cognitive Deficit Rescue in Aging mice and Tau Pathology Treatment

Anne-Cécile Guyot[1§], Charlotte Leuxe[1§], Clémence Disdier[1,2], Nassima Oumata[3], Narciso Costa[1], Gwenaëlle Le Roux[1], Paloma Fernandez Varela[4], Arnaud Duchon[5], Jean Baptiste Charbonnier[4], Yann Herault[5], Serena Pavoni[6], Hervé Galons[3], Emile Andriambeloson[7], Stéphanie Wagner[7], Laurent Meijer[8], Amie K. Lund[9] and Aloïse Mabondzo[1]*

[§] *These authors contributed equally to this work.*

Affiliations

[1]Service de Pharmacologie et d'Immunoanalyse, CEA, Université Paris-Saclay, F-91191 Gif-sur Yvette, France.

[2]Department of Pediatrics, Women & Infants Hospital of Rhode Island, The Warren Alpert Medical School, Brown University, Providence, RI, USA.

[3]University Paris Descartes, INSERM U1022, 4, avenue de l'Observatoire 75006 Paris, France

[4] Institute for Integrative Biology of the Cell: Department of Biochemistry, Biophysics and Structural Biology,CEA, Université Paris-Saclay, F-91191 Gif-sur Yvette, France.[5] Université de Strasbourg, CNRS, INSERM, IGBMC, 1 rue Laurent Fries, 67404 ILLKIRCH

[6] Service d'Etude des Prions et Infections Atypiques (SEPIA), Institut François Jacob, CEA, Université Paris-Saclay, Fontenay-aux-Roses, France.

[7]Neurofit, 67400 ILLKIRCH, France.

[8]ManRos Therapeutics, Centre de Perharidy, France.

[9]Department of Biological Sciences, Advanced Environmental Research Institute, University of North Texas, Denton, TX, USA

Corresponding author Dr Aloïse Mabondzo, Service de Pharmacologie et d'Immunoanalyse, CEA, Université Paris-Saclay, F-91191 Gif-sur-Yvette, France. Phone: 33 1 69081321; E-mail: aloise.mabondzo@cea.fr.

Figures and Legends

Fig. S1: NMR spectra of 2R)-2-[Amino-[6-(benzyl(methyl)amino)-9-isopropyl-purin-2-yl]amino]butan-1-ol (4).

Fig. S2: Brain penetrability of PDD005.

Fig. S3: PDD005 improves cognitive performance in LPS-treated mice.

Fig. S4: PDD005 improves cognitive performance in scopolamine-treated mice.

Figure S5: Full-length gels for representative western immunoblot for PHB1 and PHB2 in young adult-vehicle (n=3), aged vehicle (n=4) and aged treated with PDD005 (n= 4) corresponding to Figure 7.

Figure S6 : Full-length gels for representative western immunoblot for Tau and P-Tau in OHSCs-3x Tg- AD mice.

Figure S7: Full-length gels for representative western immunoblot for phosphorylation of GSK-3β in OHSCs in 3x Tg- AD mice. Two exposures (exposure 1: low exposure; exposure 2: high exposure) corresponding to Figure 8D

Table S1: Gene and oligonucleotide sequences for qPCR.

Table S2: Antibodies for western blot.

Table S3. PDD005 PK Parameters after Oral and i.p. Administration in WT mice.

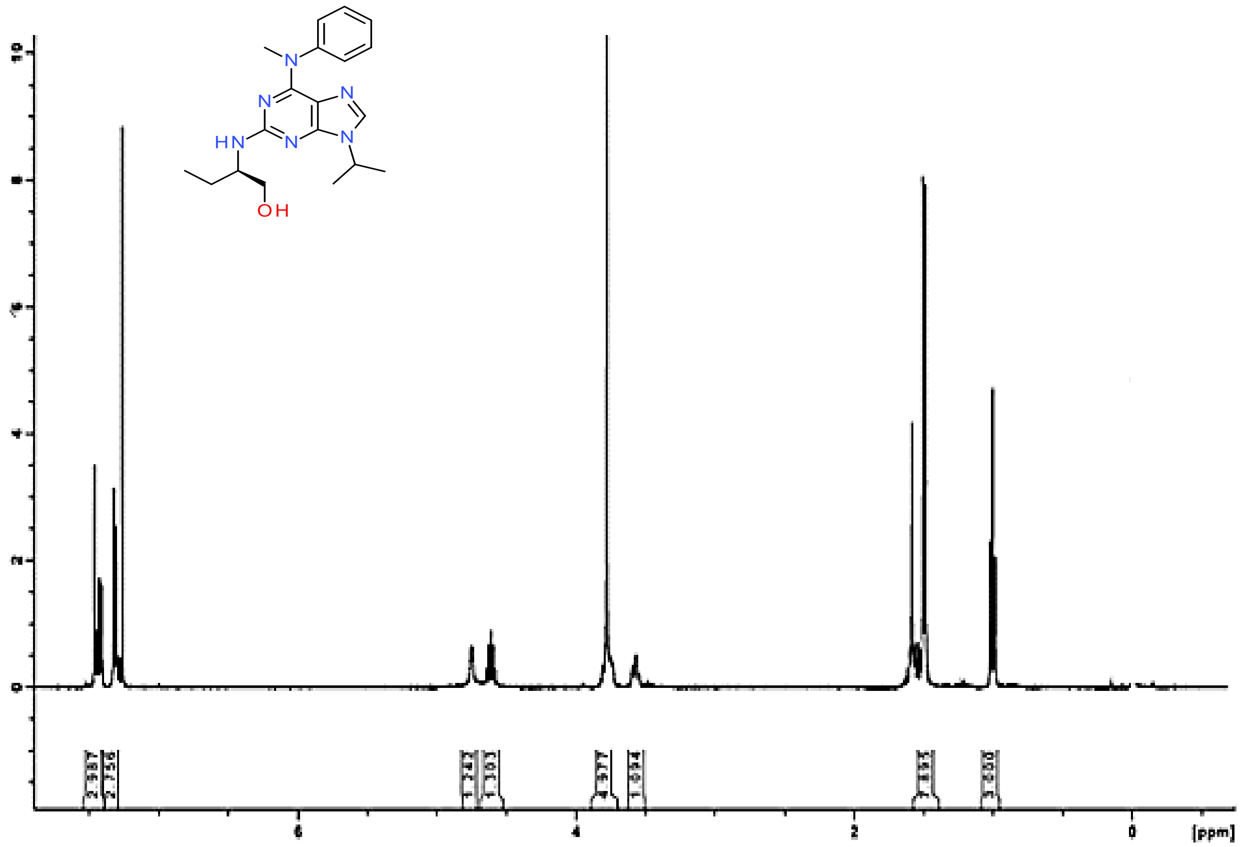

Figure S1: NMR spectra of *2R)-2-[Amino-[6-(benzyl(methyl)amino)-9-isopropyl-purin-2-yl]amino]butan-1-ol* (4).

δppm: 0.92 (t,3H, CH_3); 1.45 (d, 6H, *J*= *6.5* Hz,(<u>CH_3</u>)$_2$CH); 1.49 (m, 2 H <u>CH_2</u>CH_3); 3.50 (m, 1 H, <u>CH</u>N); 3.69 (m, 2 H <u>CH_2</u>O) ;3.72 (s, 3 H, <u>CH_3</u>N); 4.54 (hept, 1 H, $(CH_3)_2$<u>CH</u>); 4.79 (brs, 2 H, N<u>H</u>); 7.32(m, 3 H, C_6H_4) and 7.42 (m, 2 H, C_6H_4) 7.45(s, 1H, purine 8-H).

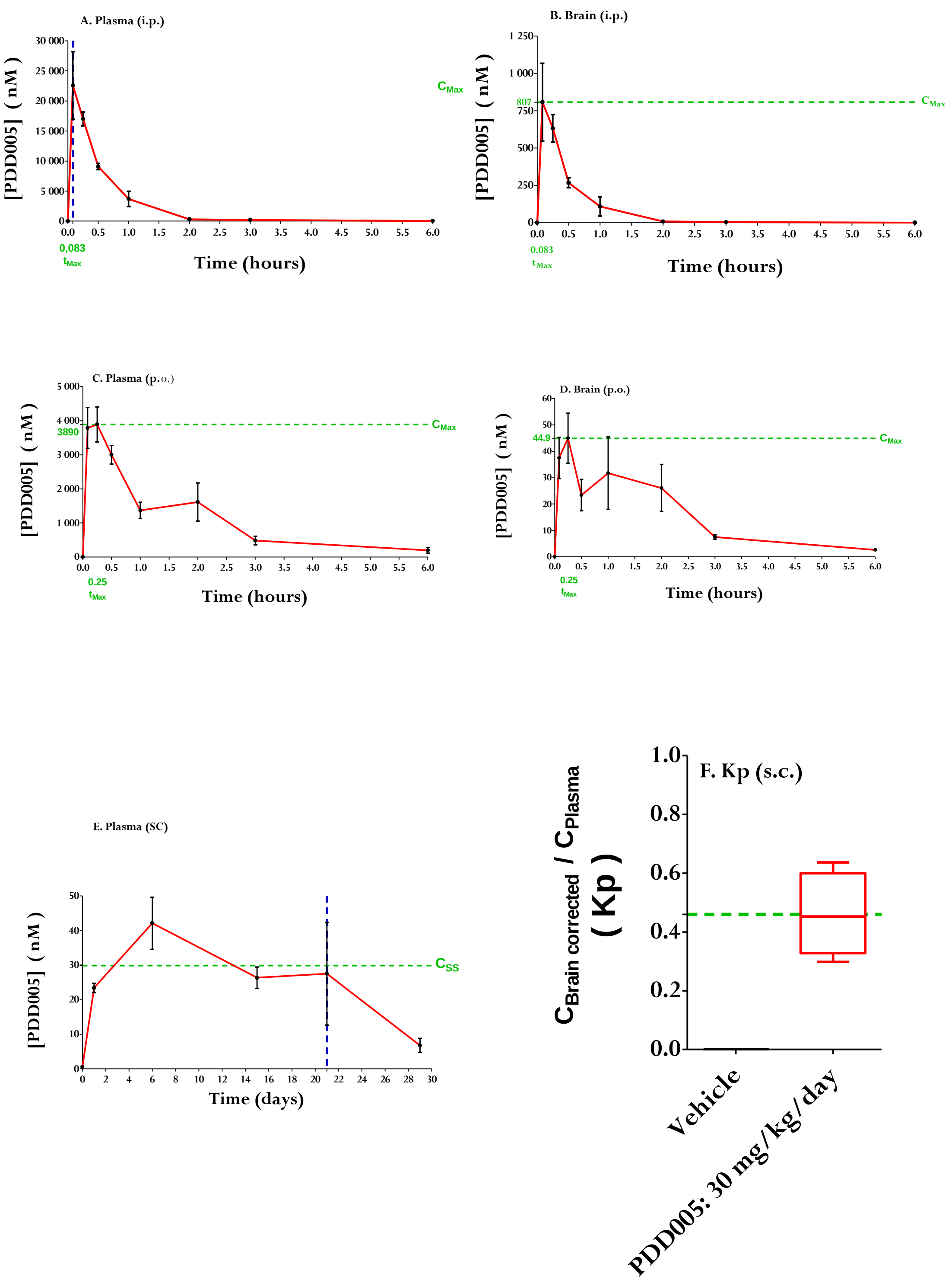

Figure S2. Translocation **of PDD005 into the CNS**. (**A**, **B)** Graph representing plasma and brain pharmacokinetic studies after single intraperitoneal administration of PDD005 in young adult WT mice. Data expressed as means ± SEM from one experiment with n = 5 mice/time point.

(C, **D)** Graph showing plasma and brain pharmacokinetic studies after single oral administration of PDD005 in young adult WT mice. Data expressed as means ± SEM from one experiment with n = 5 mice / time point. **(E, F)** Plasma concentrations and the brain/plasma partition coefficient (Kp $_{brain/plasma}$) of PDD005 after subcutaneous subchronic administration in mice showing that PDD005 crosses the BBB with good brain penetration. WT mice were exposed subcutaneously to PDD005 (30 mg/kg/day) or vehicle (DMSO/PEG/ethanol) for 28 days through an Alzet osmotic pump (Alzet ® model 2004, Q= 0.25 µL/h). Plasma was collected at different time points and, at the end of the 28 days, both brain tissue and plasma were collected for the Kp $_{brain/plasma}$ determination. PDD005 was extracted and quantified in plasma and brain tissue by LC-MS/MS. Data expressed as means ± SEM from one experiment with n = 4 mice.

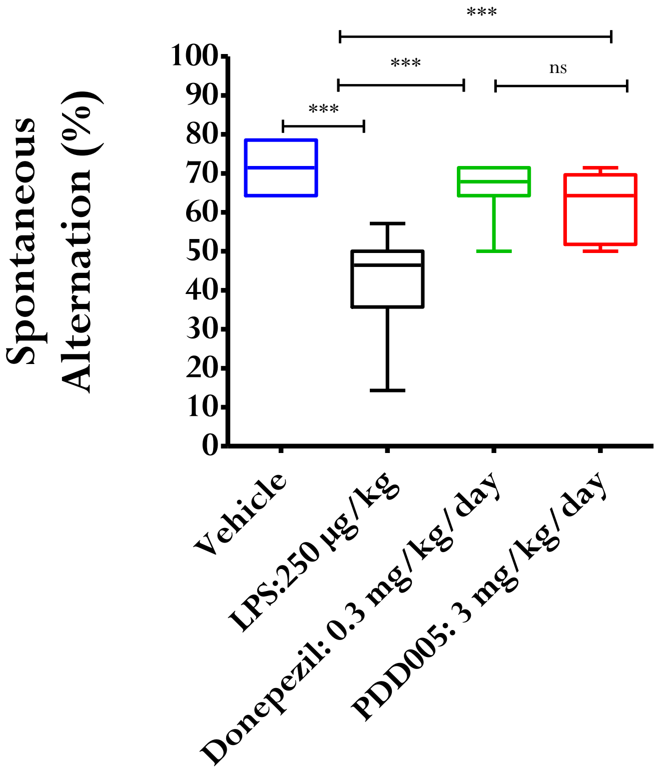

Figure S3: PDD005 improves cognitive performance in LPS-treated mice. CD-1 mice (4-5 weeks old) were used. Mice received a single intraperitoneal injection of a non-septic dose of LPS (0.25 mg/kg). The mice were then exposed to i.p. administration of PDD005 at 3 mg/kg/day or

vehicle for 7 days. Cognitive performance was then assessed by the spontaneous and continuous alternation in the T-maze. Graph showing that PDD005 rescues cognitive deficit in LPS-treated CD-1 mice. One-way ANOVA with Tukey's multiple comparison test for post hoc analysis was performed and ****P* <0.001 indicates significant differences between PDD005/LPS- and vehicle/LPS-treated CD-1 mice. Data expressed as means ± SEM with n = 8-10 mice/condition**.**

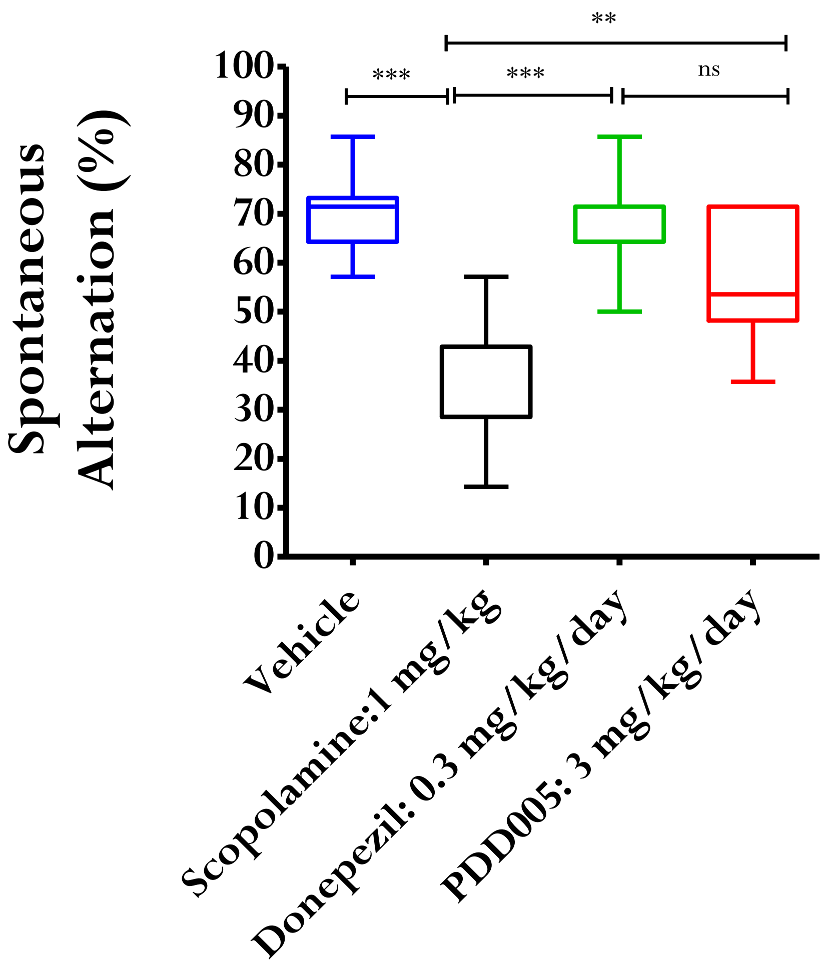

Figure S4: PDD005 improves cognitive performance in scopolamine-treated mice. CD-1 mice (4-5 weeks old) were used. Mice were treated with PDD005 at 3 mg/kg/day or vehicle by i.p. for 2 days before scopolamine administration. Cognitive/memory deficit was induced by the injection of scopolamine. Scopolamine was given i.p. at the dose of 1 mg/kg. The scopolamine injection was

adapted within a time range of 20 - 45 min before the start of the T-maze trial. Graph showing that PDD005 rescues cognitive deficit in scopolamine-treated CD-1 mice. Cognitive abilities were assessed by means of the percentage of alternation in the T-maze test. One-way ANOVA with Tukey's multiple comparison test for post hoc analysis was performed and $P < 0.05$ indicates significant differences between PDD005/scopolamine- and vehicle/scopolamine-treated mice. Data expressed as means ± SEM with n = 9-10 mice/condition**.**

Table S1: Gene and oligonucleotide sequences for qPCR

Primer	Sense	Primer Sequence	Number of base
**HPRT**	Mouse Hprt Fwd	AGT CCC AGC GTC GTG ATT AG	20
	Mouse Hprt Rev	TTT CCA AAT CCT CGG CAT AAT GA	23
**SYP**	Mouse Syp Fwd	AGT GCC CTC AAC ATC GAA GTC	21
	Mouse Syp Rev	CGA GGA GTA GTC ACC AAC	21
**PSD95**	Mouse Dlg4 Fwd	TGA GAT CAG TCA TAG CAG CTA CT	23
	Mouse Dlg4 Rev	CTT CCT CCC CTA GCA GGT CC	20
**IL-1β**	Mouse Il1b Fwd	GAA ATG CCA CCT TTT GAC AGT G	22
	Mouse Il1b Rev	TGG ATG CTC TCA GGA CAG	21

Fwd (Forward), Rev (Reverse), *HPRT* (hypoxanthine guanine phosphoribosyl transferase), *PSD95* (postsynaptic density protein 95), *SYP* (synaptophysin), *IL-1β* (interleukin-1 beta).

Table S2: Antibodies for western blot

Name	Clonality	Brand	Dilution
**Prohibitin 1**	monoclonal	Millipore	1/1000
**Prohibitin 2**	monoclonal	Millipore	1/1000
**Anti-Actin**	monoclonal	Millipore	1/2000
**Anti-Tau**	monoclonal	Millipore	1/500
**Anti-Tau (phospho T212)**	polyclonal	Abcam	1/500
**β-Actin**	monoclonal	Sigma	1/1000
**Anti-GSK-3 β**	monoclonal	BD	1/1000

**Phospho-GSK-3 β (Ser9)**	polyclonal	Cell Signaling Technology	1/500
**α-Tubulin**	polyclonal	Cell Signaling Technology	1/2500
**Anti-Mouse IgG HRP**	polyclonal	Santa Cruz BioTechnologies	1/5000
**Anti-Rabbit IgG HRP**	polyclonal	Santa Cruz BioTechnologies	1/5000
**Anti-Rat IgG HRP**	polyclonal	Jackson Immunoresearch	1/5000

Table S3. PDD005 PK Parameters after Oral and i.p. Administration in WT mice

Route	C_{max} (nM)	T_{max} (h)	$AUC_{0-\infty}$ (nMh)	$T^{1/2}$(h)
Plasma PO	3890	0.25	8111.187	1.9
Brain PO	44.93	0.25	115.25	1.8
Plasma i.p.	22564.50	0.083	13577.63	1.2
Brain i.p.	807.41	0.083	441.51	0.6

Full length gels

Figure S5: Full-length gels for representative western immunoblot for PHB1 and PHB2 in young adult-vehicle (n=3), aged vehicle (n=4) and aged treated with PDD005 (n= 4) corresponding to Figure 7.

A and B: Low exposure for PHB1 and actin, respectively

C and D; low exposure for PHB2 and actin, respectively

E High exposure for PHB2 and actin

PDD005 (30 mg/kg) and PDD2 (3 mg/Kg) was not included in the manuscript

A

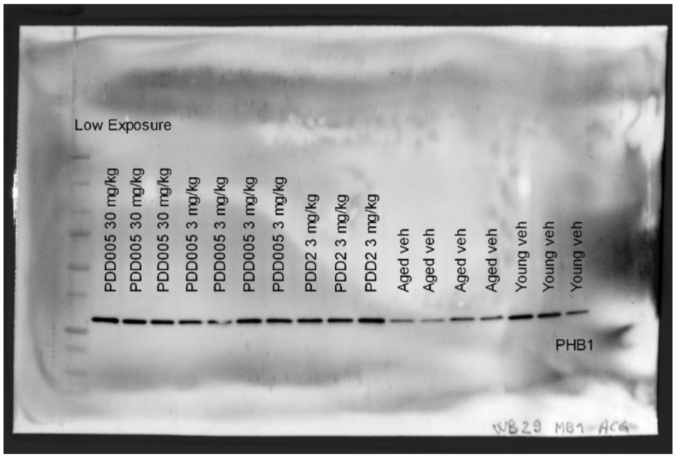

B

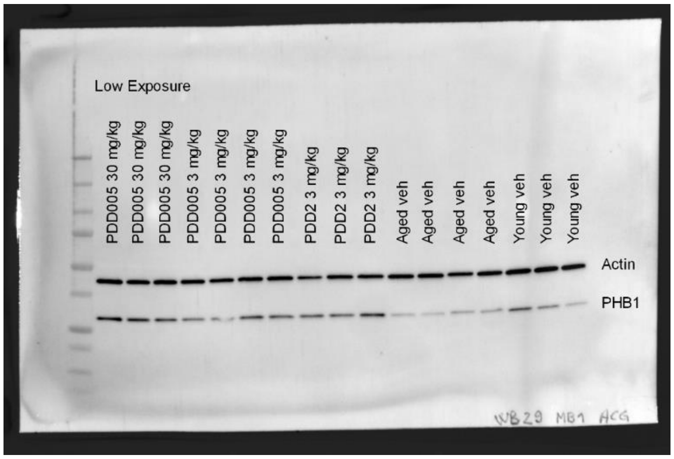

C

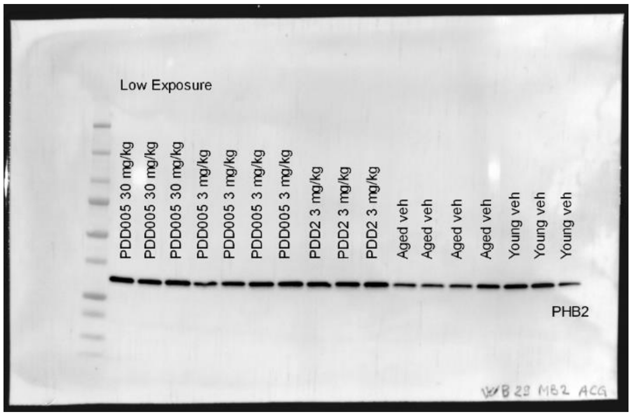
Low Exposure
PDD005 30 mg/kg
PDD005 30 mg/kg
PDD005 30 mg/kg
PDD005 3 mg/kg
PDD005 3 mg/kg
PDD005 3 mg/kg
PDD005 3 mg/kg
PDD2 3 mg/kg
PDD2 3 mg/kg
PDD2 3 mg/kg
Aged veh
Aged veh
Aged veh
Aged veh
Young veh
Young veh
Young veh
PHB2

D.

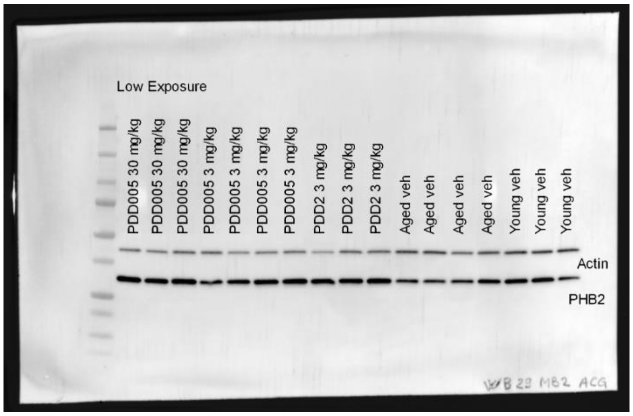
Low Exposure
PDD005 30 mg/kg
PDD005 30 mg/kg
PDD005 30 mg/kg
PDD005 3 mg/kg
PDD005 3 mg/kg
PDD005 3 mg/kg
PDD005 3 mg/kg
PDD2 3 mg/kg
PDD2 3 mg/kg
PDD2 3 mg/kg
Aged veh
Aged veh
Aged veh
Aged veh
Young veh
Young veh
Young veh
Actin
PHB2

E.

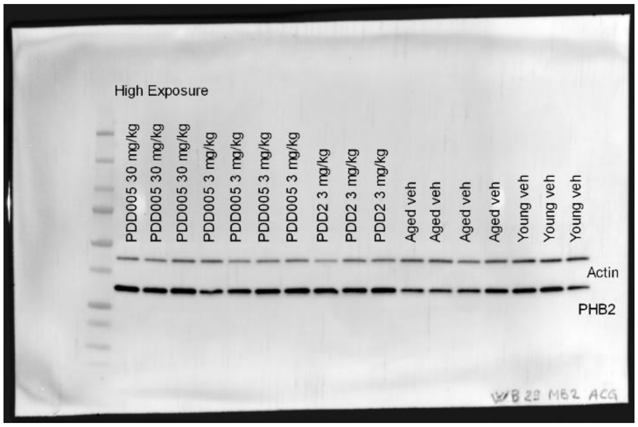
High Exposure
PDD005 30 mg/kg
PDD005 30 mg/kg
PDD005 30 mg/kg
PDD005 3 mg/kg
PDD005 3 mg/kg
PDD005 3 mg/kg
PDD005 3 mg/kg
PDD2 3 mg/kg
PDD2 3 mg/kg
PDD2 3 mg/kg
Aged veh
Aged veh
Aged veh
Aged veh
Young veh
Young veh
Young veh
Actin
PHB2

Figure S6 : Full-length gels for representative western immunoblot for Tau and P-Tau in OHSCs-3x Tg- AD mice.

Two exposures (exposure 1: low exposure; exposure 2: high exposure) corresponding to Figure 8B

Low exposure (A) and high exposure (B) for Tau
Low exposure (C) and high exposure (D) for tubulin

Low exposure (E) and high exposure (F) for Phospho Tau

Low exposure (G) and high exposure (H) for tubulin

A **B**

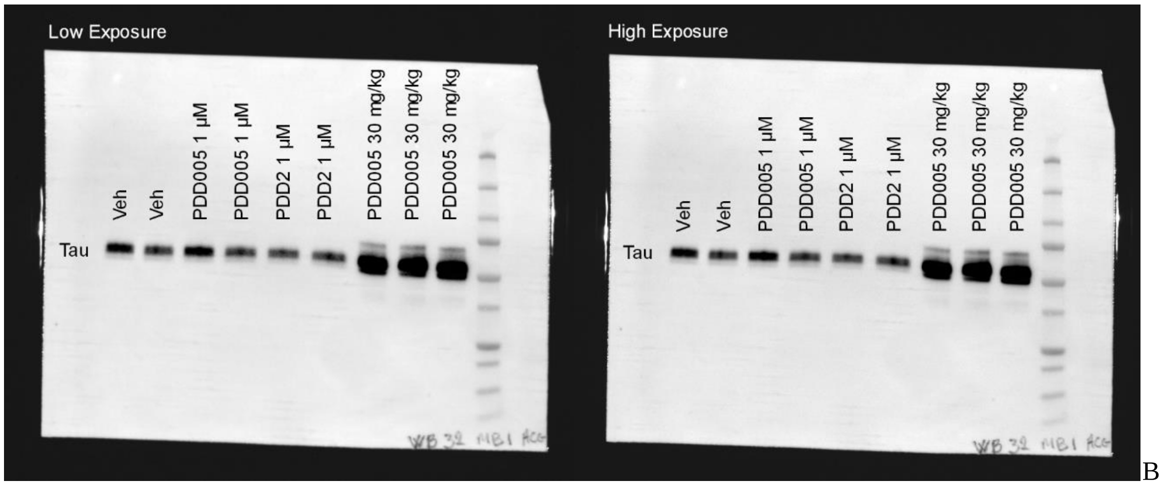

B

C

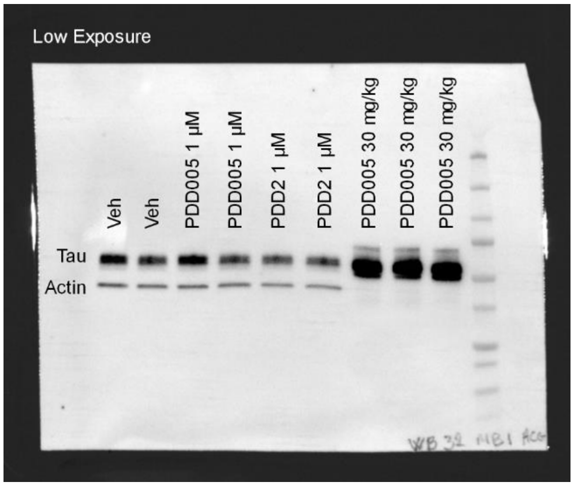
Low Exposure
Veh
Veh
PDD005 1 µM
PDD005 1 µM
PDD2 1 µM
PDD2 1 µM
PDD005 30 mg/kg
PDD005 30 mg/kg
PDD005 30 mg/kg
Tau
Actin

D

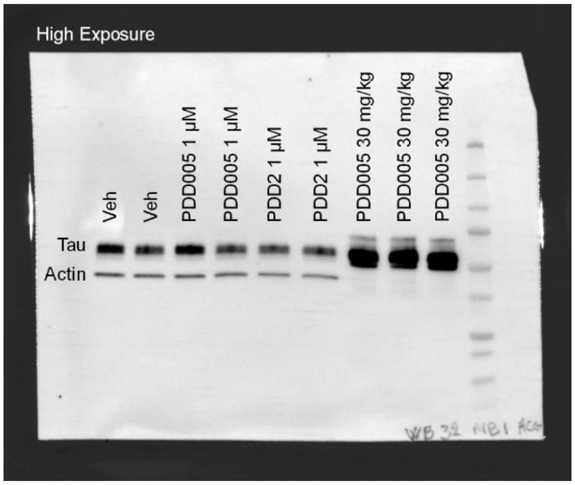
High Exposure
Veh
Veh
PDD005 1 µM
PDD005 1 µM
PDD2 1 µM
PDD2 1 µM
PDD005 30 mg/kg
PDD005 30 mg/kg
PDD005 30 mg/kg
Tau
Actin

E

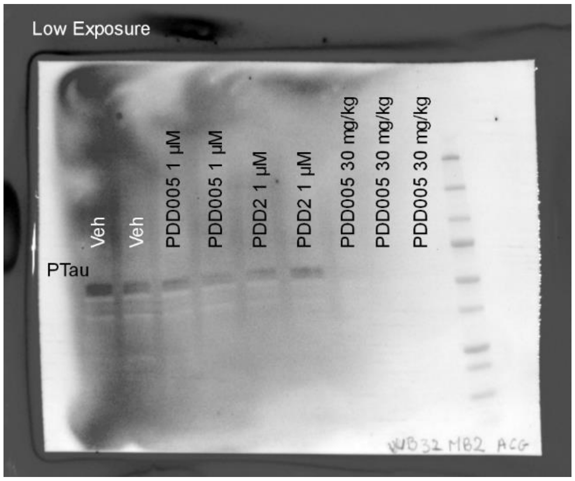
Low Exposure
Veh
Veh
PDD005 1 µM
PDD005 1 µM
PDD2 1 µM
PDD2 1 µM
PDD005 30 mg/kg
PDD005 30 mg/kg
PDD005 30 mg/kg
PTau
WB32 MB2 ACG

F

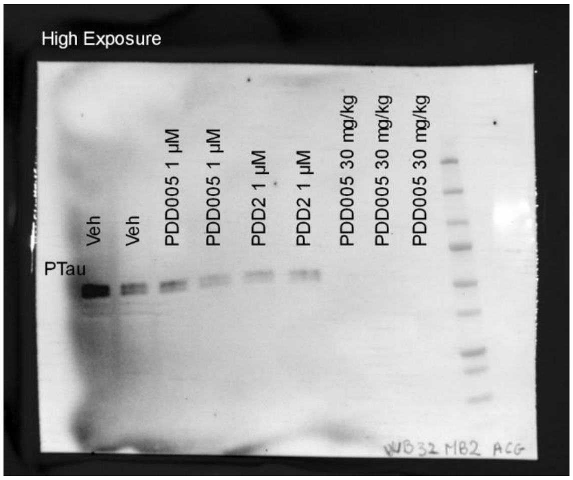
High Exposure
Veh
Veh
PDD005 1 µM
PDD005 1 µM
PDD2 1 µM
PDD2 1 µM
PDD005 30 mg/kg
PDD005 30 mg/kg
PDD005 30 mg/kg
PTau
WB32 MB2 ACG

G

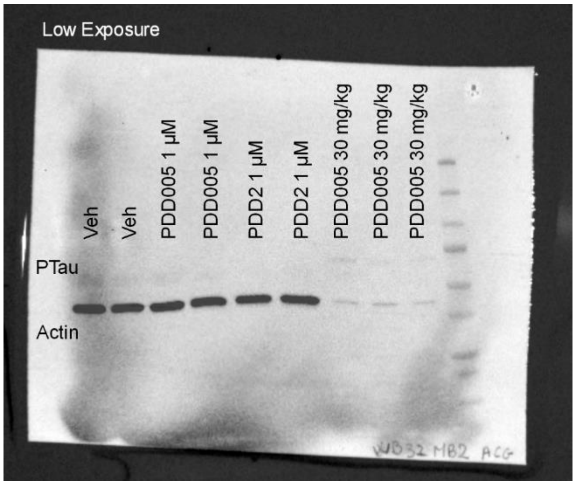
Low Exposure
Veh
Veh
PDD005 1 μM
PDD005 1 μM
PDD2 1 μM
PDD2 1 μM
PDD005 30 mg/kg
PDD005 30 mg/kg
PDD005 30 mg/kg
PTau
Actin
WB32 MB2 ACG

H

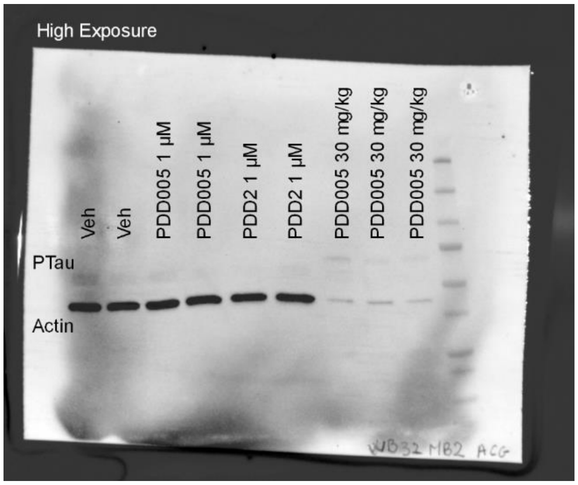
High Exposure
Veh
Veh
PDD005 1 μM
PDD005 1 μM
PDD2 1 μM
PDD2 1 μM
PDD005 30 mg/kg
PDD005 30 mg/kg
PDD005 30 mg/kg
PTau
Actin
WB32 MB2 ACG

Figure S7: Full-length gels for representative western immunoblot for phosphorylation of GSK-3β in OHSCs in 3x Tg- AD mice. Two exposures (exposure 1: low exposure; exposure 2: high exposure) corresponding to Figure 8D

A and B Low exposure (A) and high exposure (B) for GSk3β

Low exposure (C) and high exposure (D) for tubulin

Low exposure (E) and high exposure (F) for Phospho GSk3b

Low exposure (G) and high exposure (H) for tubulin

A

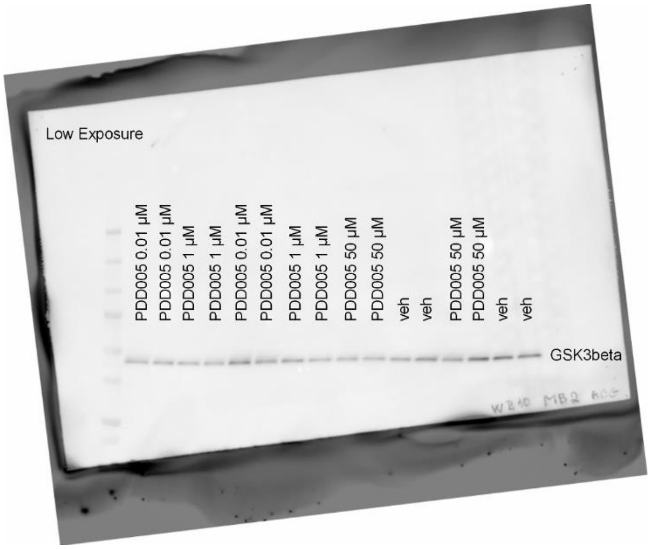

B

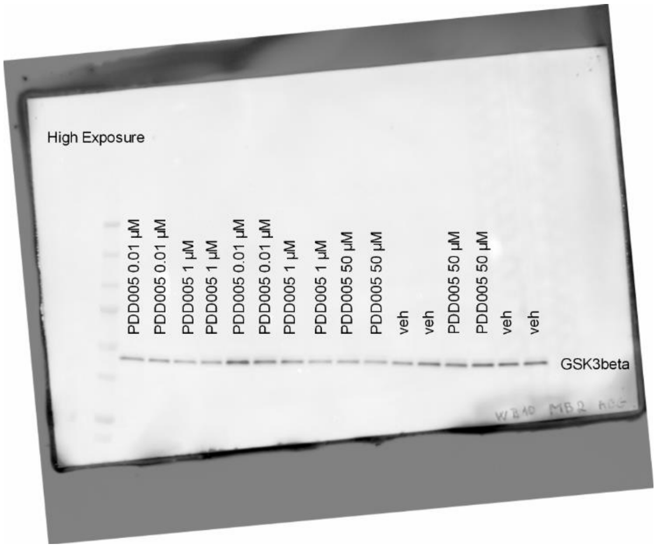

C.

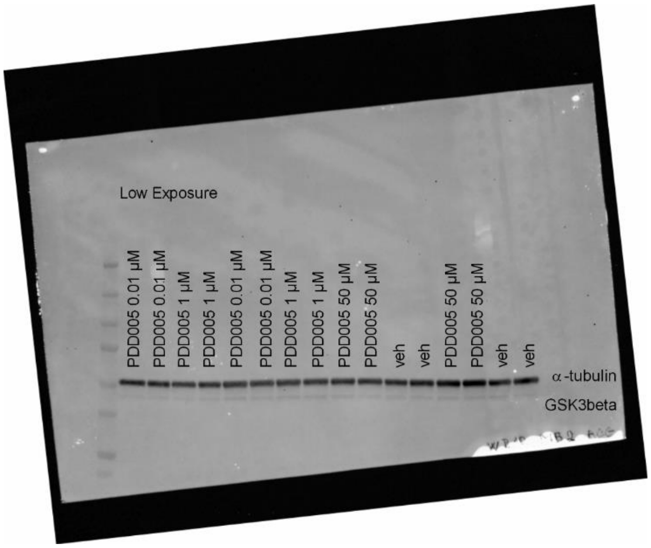
Low Exposure
PDD005 0.01 µM
PDD005 0.01 µM
PDD005 1 µM
PDD005 1 µM
PDD005 0.01 µM
PDD005 0.01 µM
PDD005 1 µM
PDD005 1 µM
PDD005 50 µM
PDD005 50 µM
veh
veh
PDD005 50 µM
PDD005 50 µM
veh
veh
α-tubulin
GSK3beta

D.

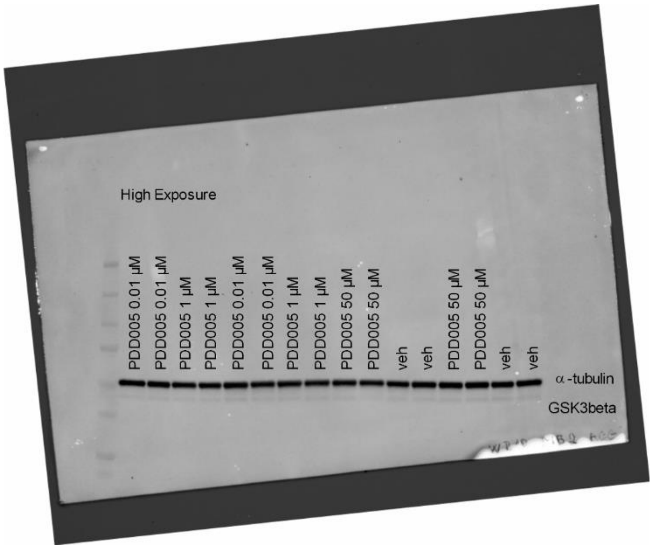
High Exposure
PDD005 0.01 µM
PDD005 0.01 µM
PDD005 1 µM
PDD005 1 µM
PDD005 0.01 µM
PDD005 0.01 µM
PDD005 1 µM
PDD005 1 µM
PDD005 50 µM
PDD005 50 µM
veh
veh
PDD005 50 µM
PDD005 50 µM
veh
veh
α-tubulin
GSK3beta

E.

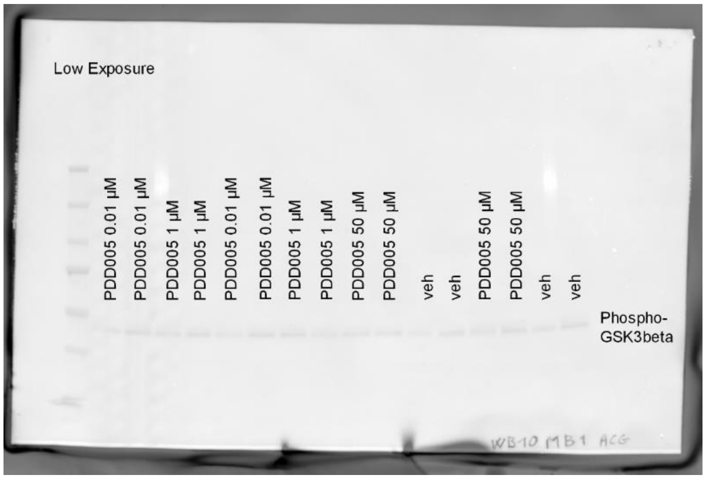
Low Exposure
PDD005 0.01 μM
PDD005 0.01 μM
PDD005 1 μM
PDD005 1 μM
PDD005 0.01 μM
PDD005 0.01 μM
PDD005 1 μM
PDD005 1 μM
PDD005 50 μM
PDD005 50 μM
veh
veh
PDD005 50 μM
PDD005 50 μM
veh
veh
Phospho-
GSK3beta

F

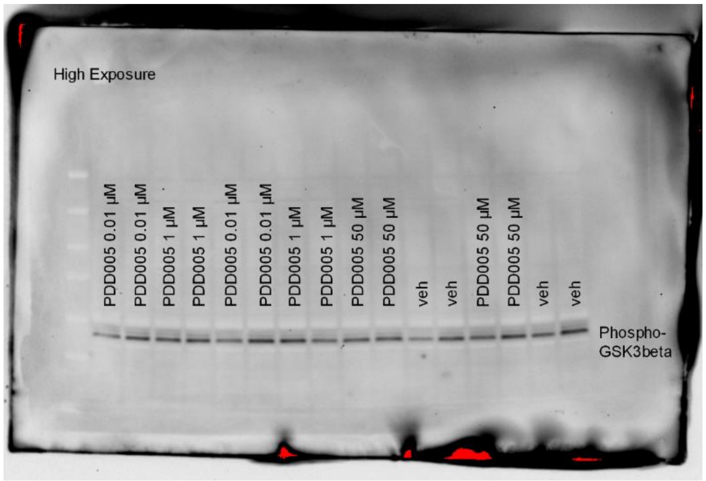
High Exposure
PDD005 0.01 μM
PDD005 0.01 μM
PDD005 1 μM
PDD005 1 μM
PDD005 0.01 μM
PDD005 0.01 μM
PDD005 1 μM
PDD005 1 μM
PDD005 50 μM
PDD005 50 μM
veh
veh
PDD005 50 μM
PDD005 50 μM
veh
veh
Phospho-
GSK3beta

G

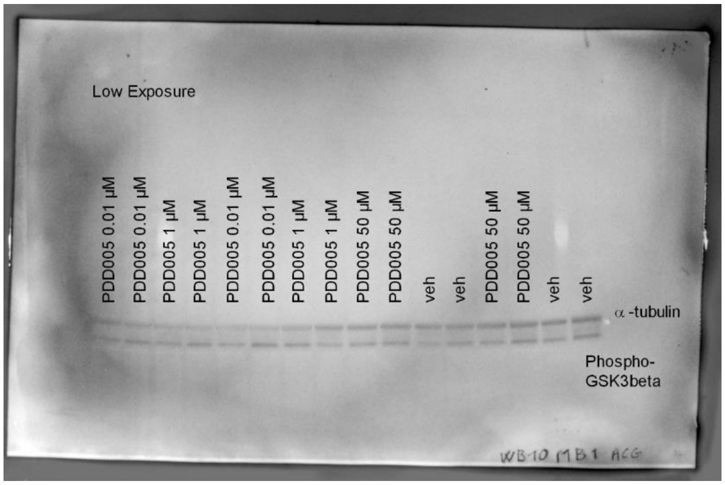
Low Exposure
PDD005 0.01 µM
PDD005 0.01 µM
PDD005 1 µM
PDD005 1 µM
PDD005 0.01 µM
PDD005 0.01 µM
PDD005 1 µM
PDD005 1 µM
PDD005 50 µM
PDD005 50 µM
veh
veh
PDD005 50 µM
PDD005 50 µM
veh
veh
α -tubulin
Phospho-
GSK3beta

H

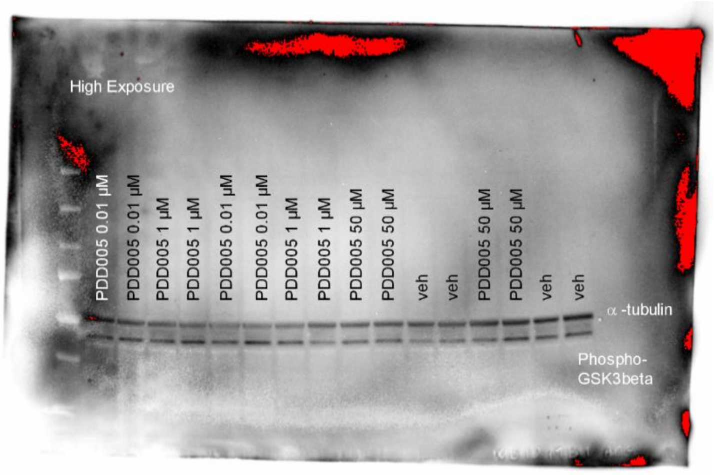
High Exposure
PDD005 0.01 µM
PDD005 0.01 µM
PDD005 1 µM
PDD005 1 µM
PDD005 0.01 µM
PDD005 0.01 µM
PDD005 1 µM
PDD005 1 µM
PDD005 50 µM
PDD005 50 µM
veh
veh
PDD005 50 µM
PDD005 50 µM
veh
veh
α -tubulin
Phospho-
GSK3beta

.
